# The Generation of Mouse and Human Huntington Disease iPS Cells Suitable for *In vitro* Studies on Huntingtin Function

**DOI:** 10.3389/fnmol.2017.00253

**Published:** 2017-08-08

**Authors:** Wojciech J. Szlachcic, Kalina Wiatr, Marta Trzeciak, Marek Figlerowicz, Maciej Figiel

**Affiliations:** ^1^Department of Molecular Neurobiology, Institute of Bioorganic Chemistry, Polish Academy of Sciences Poznań, Poland; ^2^Department of Molecular and Systems Biology, Institute of Bioorganic Chemistry, Polish Academy of Sciences Poznań, Poland

**Keywords:** Huntington disease, iPS cells, NS cells, YAC128, shRNA, huntingtin, p53, juvenile HD

## Abstract

Huntington disease (HD) is an incurable neurodegenerative disorder caused by expansion of CAG repeats in huntingtin (HTT) gene, resulting in expanded polyglutamine tract in HTT protein. Although, HD has its common onset in adulthood, subtle symptoms in patients may occur decades before diagnosis, and molecular and cellular changes begin much earlier, even in cells that are not yet lineage committed such as stem cells. Studies in induced pluripotent stem cell (iPSC) HD models have demonstrated that multiple molecular processes are altered by the mutant HTT protein and suggested its silencing as a promising therapeutic strategy. Therefore, we aimed to generate HD iPS cells with stable silencing of HTT and further to investigate the effects of HTT knock-down on deregulations of signaling pathways e.g., p53 downregulation, present in cells already in pluripotent state. We designed a gene silencing strategy based on RNAi cassette in piggyBAC vector for constant shRNA expression. Using such system we delivered and tested several shRNA targeting huntingtin in mouse HD YAC128 iPSC and human HD109, HD71, and Control iPSC. The most effective shRNA (shHTT2) reagent stably silenced HTT in all HD iPS cells and remained active upon differentiation to neural stem cells (NSC). When investigating the effects of HTT silencing on signaling pathways, we found that in mouse HD iPSC lines expressing shRNA the level of mutant HTT inversely correlated with p53 levels, resulting in p53 level normalization upon silencing of mutant HTT. We also found that p53 deregulation continues into the NSC developmental stage and it was reversed upon HTT silencing. In addition, we observed subtle effects of silencing on proteins of Wnt/β-catenin and ERK1/2 signaling pathways. In summary, we successfully created the first mouse and human shRNA-expressing HD iPS cells with stable and continuous HTT silencing. Moreover, we demonstrated reversal of HD p53 phenotype in mouse HD iPSC, therefore, the stable knockdown of HTT is well-suited for investigation on HD cellular pathways, and is potentially useful as a stand-alone therapy or component of cell therapy. In addition, the total HTT knock-down in our human cells has further implications for mutant allele selective approach in iPSC.

## 1. Introduction

Huntington disease (HD) is an incurable autosomal dominant neurodegenerative disorder caused by CAG repeat expansion in exon 1 of the huntingtin (HTT) gene (The-Huntington's-Disease-Collaborative-Research-Group, 1993). A prominent feature of HD is neuronal loss, with medium spiny neurons predominantly affected (Bates et al., 2015). Disease pathogenesis is primarily caused by the presence of mutant HTT that contains a polyQ stretch of over 40 glutamines, encoded by the CAG repeats; however, RNA toxicity might also be involved (Marti, 2016; Urbanek et al., 2016) The polyglutamine tract in the protein interferes with the physiological activity of the HTT protein, causing both loss of function and acquisition of new toxic functions (Bates et al., 2015). HTT is a multifunctional protein that is both essential in development and important for adult brain homeostasis (Wiatr et al., 2017). Mutant HTT alters multiple physiological pathways, including transcriptional regulation, signal transduction, apoptosis, intracellular vesicle trafficking, cytoskeleton assembly, and centrosome formation, making the disease pathology highly complex (Bates et al., 2015).

Despite such profound and widespread effects of mutant HTT on cellular function, disease onset usually occurs at age 30–50, and its average duration is 15–20 years (Bates et al., 2015). In rare cases of longer CAG tracts (70 or more CAG repeats), HD can develop early in life, with onset before age 20 or in childhood; such cases are called juvenile HD (Squitieri et al., 2006; Quigley, 2017). Interestingly, the process of neurodegeneration in distinct brain regions can be observed many years before the onset of motor symptoms (Tabrizi et al., 2013), even in typical HD. Although, traditionally considered a late-onset neurodegenerative disorder, a growing amount of compelling evidence has suggested that HD may be considered a neurodevelopmental disease (Wiatr et al., 2017). HTT is essential in development; lack of HTT expression results in embryonic lethality in mice at E6.5 (Duyao et al., 1995; Nasir et al., 1995; Zeitlin et al., 1995). Embryos of HdhQ111 mice, a model with mild HD features, exhibit an altered cell cycle and impaired differentiation of striatal neural progenitor cells, resulting in abnormal striatal development at E13.5–E18.5 (Molero et al., 2009). Moreover, cortical and striatal synaptic development is similarly disturbed in HD and conditional HTT knockout models (McKinstry et al., 2014). Another study has shown that the expression of mutant HTT only during mouse development is sufficient to induce HD-like phenotypes (Molero et al., 2016).

Recently, this new idea about a developmental role for mutant HTT has been strongly supported by a growing amount of research using new cellular models, including patient-derived induced pluripotent and neuronal stem cells (iPSCs and NSCs, respectively) (Mattis and Svendsen, 2015; Zhang et al., 2015; Wiatr et al., 2017). We previously demonstrated that similar molecular changes can be observed in the iPSC stage in both YAC128 mouse- and juvenile HD patient-derived cells (Szlachcic et al., 2015). The common alterations included decreased MAPK (mitogen-activated protein kinase) signaling activity and increased expression of the antioxidative protein SOD1 (superoxide dismutase 1). Finally, expression of p53 protein, which interacts with HTT and is involved in the above pathways, was decreased in both YAC128 mouse- and juvenile HD iPSCs. In addition, results from HD patient tissues and animal models demonstrate involvement of multiple signaling pathways, including the MAPK and p53 pathways, in HD pathogenesis (Bowles and Jones, 2014; Wiatr et al., 2017).

Gene silencing is one of the therapeutic strategies (Kordasiewicz et al., 2012; Miniarikova et al., 2016; Rué et al., 2016) which can potentially be used for neurodegenerative disease treatment such as cell therapy to correct patient cells or to determine how the level of mutant protein (e.g., HTT) interferes with the deregulated disease pathways. Therefore, our aim was to establish stable silencing of HTT in mouse and human HD iPS cells and subsequently to investigate the effects of HTT knock-down on deregulations of signaling pathways characteristic for HD. We designed a gene silencing strategy based on RNAi cassette in piggyBAC vector for constant shRNA expression. The HD lines with stable expression of anti HTT shRNA possess the same genetic background as the parental lines (i.e., they are isogenic) therefore another aim of isogenic line generation in the present work was the improved quality of comparison of HD phenotypes between genetically similar lines with and without stable HTT knockdown. For this we have selected the most effective HTT silencing reagents and investigated MAPK, Wnt, and p53 deregulations, which are important molecules affected in HD.

## 2. Materials and methods

This study was carried out in accordance with the recommendations of Local Ethical Commission for Animal Experiments in Poznan. The protocol was approved by the Local Ethical Commission for Animal Experiments in Poznan.

### 2.1. Mouse iPS cells culture

The HD YAC128 and WT iPSC lines were described previously (Szlachcic et al., 2015). These lines were reprogrammed using the piggyBac transposon system (Yusa et al., 2009, 2011) and were shown to be free of the reprogramming cassette after its seamless excision. Cells were cultured on gelatin-coated mitomycin C-inactivated mouse embryonic fibroblast (MEF) feeders in a medium consisting of Knockout Dulbeccos modified Eagle medium (DMEM), 15% KnockOut Serum Replacement (both Thermo Fisher Scientific, Waltham, MA), 2 mM L-Gln, 1x antibiotic antimycotic mixture, 1x MEM non-essential amino acids, 0.1 mM β-mercaptoethanol (all SigmaAldrich, St. Louis, MO), and 1,000 U/mL leukemia inhibitory factor (LIF, ORF Genetics, Kopavogur, Iceland). iPSCs were passaged with TrypLE Select (Thermo Fisher Scientific).

NSCs medium consisted of a 7:3 mixture of DMEM with Hams F12 Nutrient mix, 2% B27 supplement, 1x CTS GlutaMAX-I supplement, 1x penicillin-streptomycin (all Thermo Fisher Scientific), 5 μg/mL heparin (Sigma-Aldrich), 20 ng/mL basic fibroblast growth factor (bFGF), and 20 ng/mL epidermal growth factor (EGF; both ORF Genetics). Floating NSCs were derived from iPSCs by gentle dissociation of colonies with collagenase type IV (Thermo Fisher Scientific). The enzyme was aspirated while colonies were still attached to a plate followed by detachment with a cell scraper in DMEM/F12 plus 0.075% bovine serum albumin (BSA) Fraction V (both Thermo Fisher Scientific), and collection with a 5-mL pipette. Cell clumps were then centrifuged for 3 min at 1,300 rpm. Clumps were gently resuspended in NSC culture medium with the bFGF and EGF concentrations increased to 100 ng/mL, and the cells were seeded onto wells that had been precoated with polyHema (Santa Cruz Biotechnology, Dallas, TX, USA) to prevent adhesion. One near-confluent well of iPSCs was used for the induction of NSCs in two wells of a 6-well plate. The medium was changed every other day by allowing the spheres to settle to the bottom of a tube, after which the old medium was aspirated, and the spheres were gently resuspended in fresh medium and returned to the plates. NSCs were passaged every 4–6 days using a chopping method (Svendsen et al., 1998; Ebert et al., 2013). After 2–3 passages, bFGF and EGF concentrations were reduced to 20 ng/mL.

### 2.2. Human iPS cells culture

Human episomal HD and control iPSCs lines were previously acquired (Szlachcic et al., 2015) from public repository (NINDS Human Genetics Resource Center DNA and Cell Line Repository; https://catalog.coriell.org/1/ninds). For establishing the lines containing the stable expression of the reagents, we used HD lines with 71 CAG repeats (HD71; ND42228; derived from a 20-year-old patient), juvenile HD line with 109 CAG repeats (HD109; ND42224; derived from a 9-year-old patient), and a control line with 21 CAG repeats (ND42245). Human iPSCs were cultured in Essential 8 medium (Life Technologies) on human vitronectin-coated surfaces (VTN-N, Life Technologies) and were passaged using 0.5 mM EDTA in PBS.

### 2.3. Construct and isogenic line derivation

**Figure 1:**
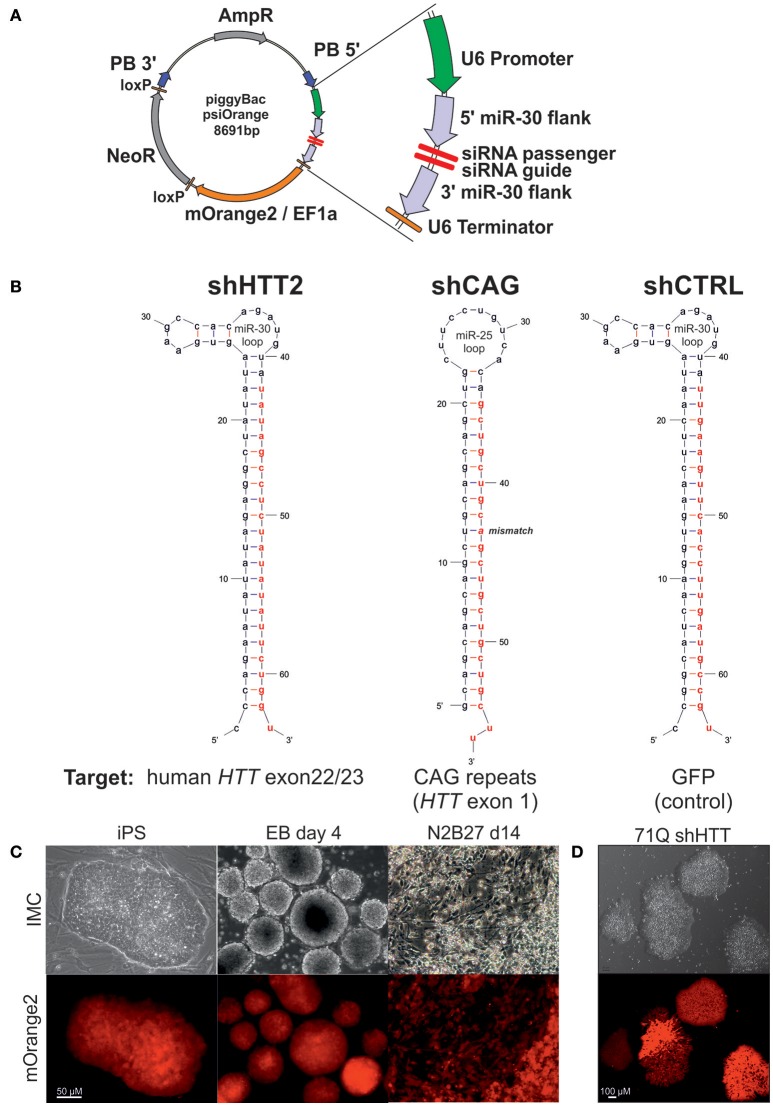
The silencing cassette design and derivation of the iPSC lines. **(A)** Schematic of the psiOrange vector. The silencing cassette is inserted into the iPSC genome as a piggyBac (PB) transposon bordered by 5′ and 3′ PB arms. An shRNA is expressed under regulation of a U6 promoter and is flanked by pri-miR-30 5′ and 3′ sequences, which are 151 and 128 bp long, respectively. Additionally, the mOrange2 fluorescent reporter and NeoR resistance genes are included within the transposon. **(B)** Schematic of shRNA sequences. Effector guide strands are marked in red. **(C)** mOrange reporter expression is sustained in the mouse iPSC state and upon differentiation as embryonic bodies (EB) or neuroectoderm (N2B27 conditions). **(D)** mOrange reporter is expressed in human iPSCs with shRNA cassettes.

Constructs (Figure 1A) composed of a U6 promoter, a miR-30 5′ flank (151 bp), an shRNA sequence, a miR-30 3′ flank (128 bp), a U6 terminator (TTTTTT), an EF1alpha promoter, an mOrange2 reporter gene, and an SV40 pA site were were synthesized by Genscript (Piscataway, NJ) and cloned into a pPB-HKS-neoL vector obtained, by removing the EGFP reporter gene, from a pPB-UbC.eGFP-neo plasmid (Yusa et al., 2009). The shRNA sequences (Figure 1B, Table S1) targeting human huntingtin (shHTT) and EGFP (control reagent, shCTRL) were designed using the RNAi Codex database (Olson et al., 2006) with a mir-30 loop between the passenger and guide strands. The allele-specific shCAG reagent targeting the CAG tract in mutant HTT was adapted from ref. (Fiszer et al., 2013), along with the miR-25 loop. To generate cell lines stably expressing the shRNA construct, 0.56 x 10^6^ cells from two iPS lines derived from YAC128 animals were electroporated with 10 μg of the piggyBac transposase-encoding plasmid (hyPBase) (Yusa et al., 2011) and 2 μg of each shRNA plasmid in HEPES-buffered DMEM. Cells were seeded in K15 medium and selected on G418 (300 μg/mL) (Thermo Fisher Scientific) for 8 days. For derivation of clonal lines, after another 7 days without selection, colonies expressing the mOrange2 reporter gene were picked and expanded. In the case of human iPSCs, the cells were gently detached in clumps containing several cells. For each electroporation, 1/3 well of a confluent 6-well plate was used. The same plasmid concentration, electroporation, and selection protocols were used as for mouse cells. After the antibiotic selection all cells were mOrange2 positive and were passaged after reaching confluence. Material for protein expression analysis was collected after at least three passages.

### 2.4. PCR genotyping

For genotyping, DNA was isolated using a Spin Column Genomic DNA Kit (Bio Basic Inc., Markham, Canada), and GoTaq G2 polymerase (Promega GmbH, Mannheim, Germany) was used for PCR. Genotyping to confirm insertion of the shHTT and shGFP constructs was performed using multiplex PCR with a set of primers specific for the YAC128 transgene [intron 26–27 of human HTT; forward (F): 5′-CCTCTTATATATGGATGCTAATCTCATTC-3′ and reverse (R): 5′-AATACACAACACATGAGAGCATATAGAAC-3′] as the internal control, and primers specific for the construct. The forward, universal primer (U6: 5′-CGGCAGCACATATACTAGTCGA-3′) was designed to be in the U6 promotor-miR30 boundary, while the reverse primers were specific for each construct (shHTT: 5′-GCCTCTATATATTCTGGGCGCT-3′, shCTRL: 5′-GAAGTTCACCTTGATGCCGG-3′). The genotyping analyses were performed using Touchdown PCR with the following cycling conditions: 3 min at 94°C; 12 x (35 s at 94°C, [45 s at 64°C - 0.5°C/cycle], and 45 s at 72°C); 25 x (35 s at 94°C, 30 s at 58°C, and 45 s at 72°C); and finally, 2 min at 72°C. Genotyping for the CAG-composed shCAG construct was conducted using two pairs of primers in separate reactions: pair 1 with the universal U6 forward primer and the shCAG-specific reverse primer (A2_R: 5′-TGTGACAGGAAGCAGCTGC-3′); and pair 2 with the shCAG-specific forward primer (A2_F: 5′-CTGCTGCTGCTTTGCCTACT-3′) and the universal EF1a-promoter specific primer (EF1a: 5′-GGGGCGAGTCCTTTTGTATGA-3′). Standard PCR cycling was used for these reactions. Reaction products were separated on 1.3% agarose gels in TBE buffer and were visualized using ethidium bromide.

### 2.5. ERK activation assay

The ERK assay in iPSCs was performed as described previously (Szlachcic et al., 2015). Briefly, the day before the start of experiments, the medium was exchanged for serum-free medium without LIF, and the cells were starved for 24 h. Then, without changing the medium, 20 ng/mL bFGF was added, and the cells were incubated for 5, 10, or 30 min. After each incubation period, the medium was quickly discarded, and the cells were immediately lysed using a protein-lysis buffer. As NSC culture media containing bFGF and NSCs depend on the MAPK signaling pathway, the basal levels of pERK1/2 were measured in cell lysates taken directly from cultures.

### 2.6. Western blotting

For protein isolation, the cells were washed using PBS, lysed in a protein-lysis buffer containing 60 mM Tris base, 2% SDS, 10% sucrose, 2 mM PMSF, and 1x Halt Phosphatase Inhibitor Cocktail (Thermo Scientific), and then homogenized. An aliquot of 20–30 μg of total protein per lane was dissolved in loading buffer containing 2-mercaptoethanol and was then boiled for 5 min. The proteins were separated using SDS-PAGE (5/10% stacking/resolving gels) and Laemmli buffer. For comparison of NS WT vs HD cell lines, we used 10% TGX Stain-free FastCast Acrylamide gels (Bio-Rad, Hercules, CA, USA). HTT was separated in 4% stacking/5% resolving gels using commercial XT Tricine running buffer (Bio-Rad). The proteins were semi-dry-transferred (Transblot Turbo, Bio-Rad) to nitrocellulose or PVDF (huntingtin) membranes and the blots were blocked using 5% nonfat milk in TBS-Tween. Blots were subsequently incubated overnight at 4°C with primary antibody diluted in TBS-Tween containing 5% milk or BSA. The antibodies used were purchased from Cell Signaling (Danvers, MA) unless otherwise stated and were as follows: rabbit anti-β-catenin (1:1,000, cat. 8480); rabbit anti-phospho-β-catenin (Ser33/37) (1:1,000, cat. 2009); rabbit anti-p44/42 MAPK (ERK1/2) (1:2,000, cat. 4695); rabbit anti-phospho-p44/42 MAPK (ERK1/2) (Thr202/Tyr204) (1:1,000, cat. 4370); mouse anti-p53 (1:1,000, cat. 2524); rabbit anti-p53 (DO-1, 1:600, Santa Cruz, sc-126); mouse anti-phospho-p53 (S15) (1:1,000, cat. 9284); mouse anti-OCT3/4 (1:1,000, Santa Cruz, sc-5279); mouse anti-nestin [Rat-401 (Hockfield and McKay, 1985), 1:100; DSHB, Iowa City, IA]; rabbit anti-PAX6 (1:1,000; Millipore, Billerica, MA; AB2237); rabbit anti-SOX1 (1:1,000, cat. 4194); mouse anti-TUBB3 [6G7 (Halfter et al., 2002), 1:100, DSHB]; anti-huntingtin antibodies: mouse MW1 (Ko et al., 2001) (1:1,000, DSHB), 4–19 (Macdonald et al., 2014) (1:1,000; CH00146, CHDI Foundation, Corriel Cell Repositories), 3–16 (1:1,000; Sigma-Aldrich; H7540), and MAB2166 (1:2,000, Millipore); and mouse anti-GAPDH (1:10,000, Millipore, MAB374). The blots were then incubated for 2 h at RT with HRP-conjugated secondary antibodies raised against rabbit or mouse antibodies (1:2,000–1:20,000 dilution, Jackson ImmunoResearch, West Grove, PA), and the labeled bands were detected using the ECL-based WesternBright Quantum (Advansta Inc., Menlo Park, CA) or homemade ECL reagent. Data was collected using ChemiDoc XRS+ System with Image Lab v5.2 Software (Bio-Rad). To avoid overexposure of any band, image acquisition times were set based on image histograms. Images were not processed before quantitation. All analyses were performed as three independent technical replicates. Data within a gel were normalized to GAPDH or total protein (WT vs, HD NSC analyses), and data between gels were normalized to the average of WT or isogenic shGFP samples.

### 2.7. Immunostaining

For immunostaining, the cells were cultured in 24-well dishes on gelatin- and feeder cell-coated coverslips. The cells were washed using PBS, fixed by incubation with 4% paraformaldehyde for 15 min at RT, washed, and permeabilized using 0.3% Triton in PBS for 10 min at RT. Blocking was performed in 3% BSA, 0.1% Tween-20 in PBS for 30 min at RT, and the primary-antibody incubation was conducted overnight at 4°C in an antibody dilution solution composed of 5% normal serum of secondary antibody species (Jackson Immunoresearch) and 0.1% Tween-20 in PBS. The primary antibodies used were as follows: anti-OCT3/4 (1:500, Santa Cruz, sc-5279), rabbit anti-nestin (1:400, Abcam, ab27952), mouse anti-nestin (1:50, DSHB, Rat-401), rabbit anti-PAX6 (1:50, Millipore, AB2237), rabbit anti-SOX1 (1:100, cat. 4194), and mouse anti-TUBB3 (Tuj1) (1:400, Millipore, MAB1637). After washing with PBS, the cells were incubated for 1 h at RT with a proper Cy3- or AlexaFluor488-conjugated secondary antibody (1:500, Jackson Immunoresearch) in the antibody dilution solution. A 5-min incubation in DAPI (1:10,000) dissolved in water was used for counterstaining. Additionally, the primary antibodies were omitted in the secondary antibody controls. The coverslips containing the cells were mounted on slides using anti-fade glycerol/propyl gallate mounting medium. The specimens were analyzed using a DMIL LED inverted fluorescence microscope (Leica Microsystems, Wetzlar, Germany) and Leica Application Suite Software. Confocal microscopy was performed using a Leica TCS SP5 microscope.

### 2.8. Statistics

Two-group comparisons of the gene expression data were conducted using the unpaired Students *t*-test. The data for ERK1/2 activation in iPSCs were subjected to a two-way ANOVA, followed by Bonferroni *post-hoc* tests. Pearsons simple correlation was used to determine relationships between mutant HTT and other analyzed protein expression levels. *P*-values of less than 0.05 were considered significant. Whiskers in box plots represent 5–95 percentile, while error bars on bar graphs are presented as SEM.

Full description of methods is provided in the Supplementary Materials online.

## 3. Results

### 3.1. Generation of mouse YAC128-HD-iPSCs and human HD109, HD71, and control iPS isogenic cell lines with stable expression of shRNA targeting mutant HTT

Cell lines with continuous expression of RNAi constructs that effectively silence target genes can be used as tools in cell therapy and for the generation of shRNA isogenic lines to specifically assess the effect of mutant HTT on early HD phenotypes. We have assembled a silencing construct and stably integrated it into the iPSC genome; this construct is based on the piggyBac transposase system (Yusa et al., 2011) and contains anti-HTT or control shRNA in the mir-30 backbone (Paddison et al., 2004), and the gene encoding mOrange2 fluorescent protein (Shaner et al., 2008) as a reporter (Figure 1A). To establish mouse isogenic iPSC, we used our previously generated HD iPSC lines (Szlachcic et al., 2015) derived from YAC128 mice (Slow et al., 2003) and several shRNA silencing constructs. Using the constructs, we first evaluated the efficiency of 3 anti-HTT shRNAs (shHTT1-3) in iPSCs (see next section, Table S1). Then, we used the most effective reagent (shHTT2), as well as a reagent specifically targeting the CAG repeats (shCAG) (Fiszer et al., 2013) or targeting EGFP as a control (shCTRL), and we generated 12 isogenic iPSC lines expressing these shRNAs from two HD iPSC lines (two clones per line and reagent; Figure 1B, Table S1). The lines were genotyped for the presence of a proper shRNA construct (Figure S1). We also established human HD iPSCs expressing the sHTT2, shCAG, or shCTRL from HD109 (109 CAG repeats), HD71 (71 CAG repeats), and Control (21 CAG repeats) iPSC lines.

Floating neurospheres (non-adherent neural stem cells, NSCs) with and without the reagents were generated by iPSC differentiation and expressed characteristic cellular markers (Figures S2A–E). To investigate survival of cells containing reagents in the mouse brain, we injected the cells into the mouse striatum, and using the PACT method (Yang et al., 2014) we found that they survived for the 8-week test period (Figure S2F). The mOrange2 reporter exhibited a strong red fluorescent signal in pluripotent shRNA iPSCs, embryoid bodies and throughout adherent differentiation (Figures 1C,D). Summarizing, we have generated both mouse and human HD lines containing construct with several shRNA reagents targeting various parts of mRNA for human HTT able to differentiate to NSC and able to survive in mouse brain upon delivery by injection.

### 3.2. Continuously expressed shRNA reagents can efficiently silence mutant HTT in mouse iPSCs and NSCs

**Figure 2:**
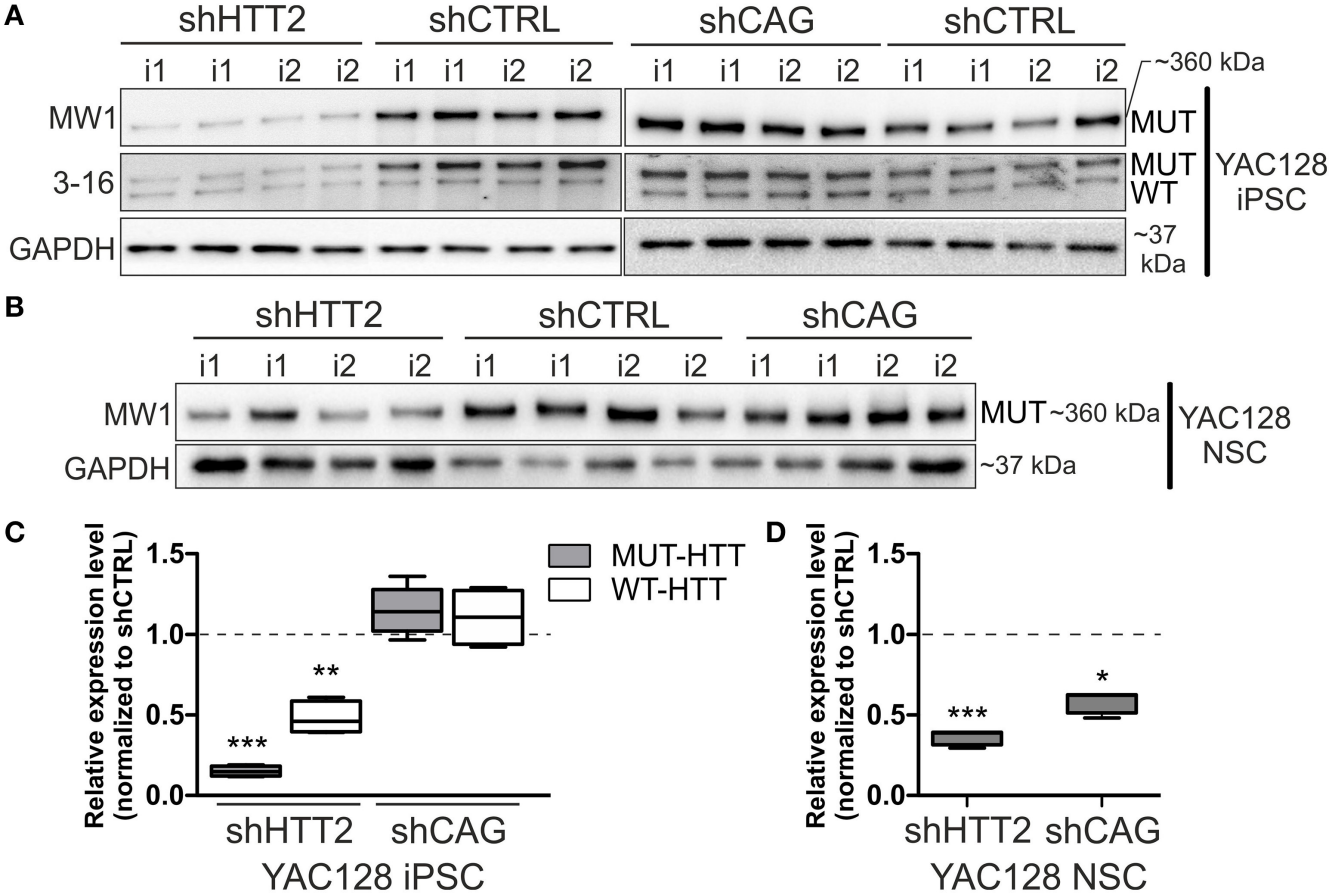
Isogenic YAC128 iPSC and NSC lines with efficient shRNA-mediated silencing of mutant HTT. **(A,B)** Western blot analysis reveals efficient mutant HTT silencing in iPSC lines with shHTT2 but not shCAG reagent, compared to shCTRL lines. **(C,D)** Mutant HTT is continuously silenced by the shHTT2 reagent after iPSCs differentiating into the NSC state, as assessed by western blots. However, the shCAG reagent changes its mode of action, decreasing mutant HTT expression in NSCs. ^*^p < 0.05, ^**^p < 0.01, ^***^p < 0.001; *N* = 4 lines for each reagent for both iPSC and NSC analysis (the same lines were used); i1, i2 isogenic lines derived from separate parental lines 1 and 2. In (**A**, iPSCs) blots were cropped; full-length blots are presented in Figure S8.

We first evaluated efficiency of three anti-huntingtin shRNA reagents (shHTT1, shHTT2, and shHTT3) in mouse HD iPSCs without clonal selection. shHTT1 and shHTT3 reagents lowered levels of mutant huntingtin by 54 ± 8% (*p* = 0.0042) and 35 ± 7% (*p* = 0.004), respectively (Figure S3), as assessed by western blotting. The most efficient reagent, shHTT2, which lowered HTT expression up to 85% was used in further studies and derivation of clonal mouse HD iPSC lines. The lines containing shHTT2, shCAG, or shCTRL reagents were tested for the expression of mutant and normal HTT. Western blotting with polyQ-specific antibody revealed that HTT was effectively silenced in iPSC lines containing the shHTT2 reagent (−85 ± 3%, *p* = 0.0043; shHTT2 vs. shCTRL; Figures 2A,C). HTT was not silenced, and in some cases was upregulated, in lines containing stable expression of the shCAG reagent; however, the upregulation was non-significant (19.5 ± 13%, *p* = 0.2; shCAG vs. shCTRL). We also analyzed the effects of shRNA reagents on expression of wild-type mouse HTT. Its expression was reduced in shHTT2-iPSC lines (−53 ± 13%; *p* = 0.032) but was unchanged in shCAG-iPSC lines.

Next, we assessed whether the effect of HTT silencing with shRNA reagents was preserved after differentiation from iPSCs into a neural lineage. Therefore, we differentiated iPSCs containing shHTT2 to the state of non-adherent NSCs in bFGF and EGF conditions (Figure S1). Similar to iPSCs, mutant HTT was also effectively silenced in shHTT-NSC lines but with a slightly lower efficiency (−62 ± 19%, *p* = 0.0005; shHTT vs. shCTRL; Figures 2B,D). Surprisingly, the shCAG reagent, which was previously ineffective in iPSCs, became effective in the NSC state and decreased mutant HTT protein levels by 40 ± 10% (*p* = 0.01; shCAG vs. shCTRL). Summarizing, we have selected a shHTT2 reagent which is suitable for continuous expression iPSC and evokes stable silencing of mutant HTT with high efficiency in mouse cells.

### 3.3. Stable expression of shRNA reagents silenced total HTT in human HD cells

**Figure 3:**
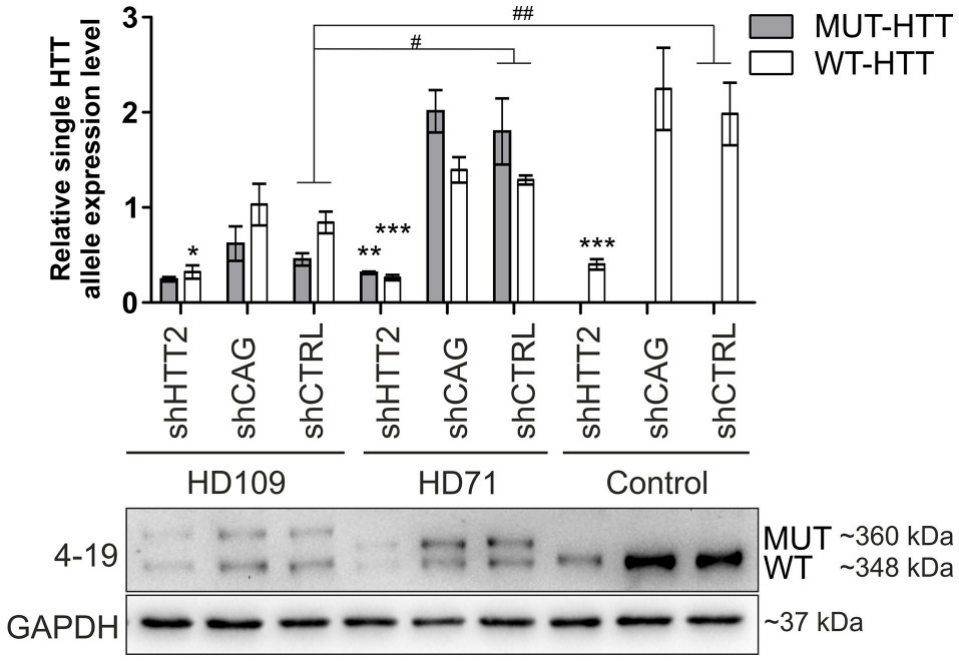
Huntingtin can be effectively and stably silenced with shRNAs in human HD and control lines Western blot analysis of mutant (MUT) and wild-type (WT) huntingtin protein expression in iPSC lines from patients with 109 CAGs (HD109) and 71 CAGs (HD71) and healthy controls (Control, 21 CAGs) with shRNA reagents reveal efficient silencing with the shHTT2 reagent. Despite differences in initial alleles expression levels between cell lines the reagent lowered both alleles to similar levels. ^*^Statistically significant difference vs. isogenic shCTRL line; #Statistically significant difference vs. Control shCTRL line; ^*^ and #*p* < 0.05; ^**^ and ##*p* < 0.01; ^***^p < 0.001. Note that the results for Control lines were normalized to reflect the level of one HTT allele (densitometry values of detected HTT level in control lines was divided by 2). For each patient, one cell line per each reagent was used (9 modified cell lines in total).

Human HD109, HD71, and Control iPSC lines expressing the HTT targeting shHTT2 reagent revealed effective silencing of both mutant (HD109: −51 ± 22%, *p* = 0.059; HD71: −83 ± 21%, *p* < 0.01) and normal HTT (HD109: −62 ± 18% *p* < 0.05; HD71: −79 ± 4% *p* < 0.001; Control: −80 ± 17% *p* < 0.001; Figure 3). In addition we have also tested the total level of HTT mRNA and found its effective silencing (Figure S4). Similarly to mouse HD iPSCs, the shCAG reagent was ineffective in human iPSC lines. We have also noticed major differences in expression levels of mutant and normal HTT which seemed to be dependent on CAG length in human iPSC lines. The expression level of normal HTT and total HTT was most significantly decreased in HD109 (normal allele: −63 ± 17.5% *p* < 0.05 as normalized to a single allele; total HTT level: −72 ± 16%, *p* < 0.01; HD109 vs. Control) while the level of normal HTT have revealed the trend toward decreased HTT level in HD71 (normal allele: −39 ± 17% *p* = 0.0599 as normalized to a single allele; total HTT level: −25 ± 17% *p* = ns; HD71 vs. Control). Moreover, HD109 had lower protein expression level of both HTT alleles as compared to HD71 lines (mutant allele: −78 ± 21%, *p* < 0.01; normal allele: −40 ± 10% *p* < 0.05; total HTT: −62 ± 15% *p* < 0.05). Regardless of the basal level of the HTT, the level of both mutant and normal allele dropped to comparable levels after silencing with shHTT2 in all lines. Therefore, some border level of HTT protein remains after silencing by a given shRNA and is independent of the initial level of the HTT. The low level of the normal HTT in HD109 iPSC is distinct to the much higher normal mouse HTT level in mHD-YAC128 iPSC (Figure S5). In summary, the silencing of HTT in human cells was efficient with shHTT2 reagent however the experiments additionally revealed a general decrease in expression of both mutant and normal HTT in HD109 iPSC vs. Control iPSC.

### 3.4. The effects of HTT silencing on Wnt and ERK signaling in HD shRNA-iPS cell lines

**Figure 4:**
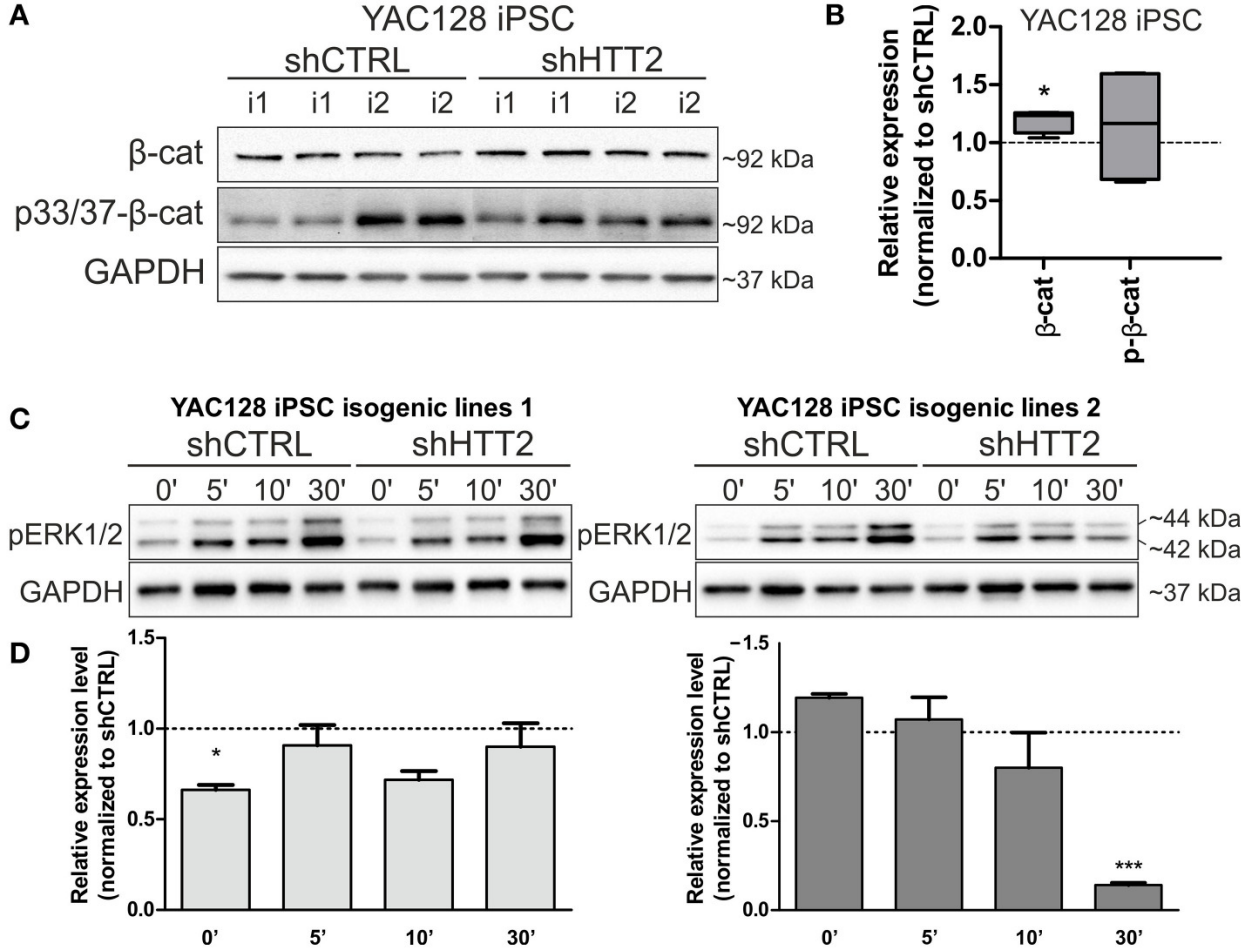
Effects of mutant HTT knockdown on MAPK and Wnt pathways in mouse iPSCs. **(A,B)** Western blot analysis of shHTT2 effects on the Wnt pathway revealed a significant increase in total β-catenin levels but not phospho-β-catenin (S33/37) levels. *N* = 4 lines for each reagent; **(C,D)** Isogenic lines (*N* = 2 for each reagent) originating from separate parental lines (1 and 2) show different responses to bFGF-induced activation of ERK1/2 phosphorylation (Thr202/Tyr204) after 30 min of stimulation. ^*^p < 0.05, ^***^p < 0.001. In Panel **(A)** blots were cropped; full-length blots are presented in Figure S8.

**Figure 5:**
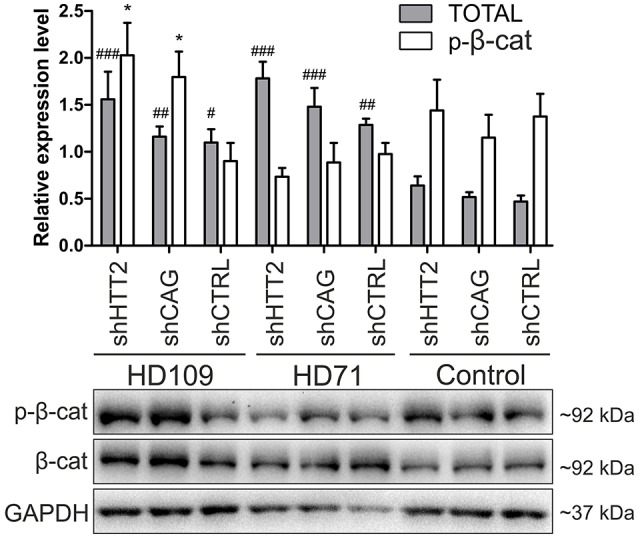
Effects of mutant HTT knockdown on Wnt pathway in human iPSCs. Western blot analysis of shHTT2 effects on the Wnt pathway revealed a significant increase in total β-catenin levels in HD109 and HD71 lines as compared to healthy Control lines, but no effects of shRNA reagents. Phospho-β-catenin (S33/37) did not differ between lines with different CAG numbers, however its level was affected by both effective shHTT2 and ineffective shCAG reagents in HD109 iPSCs only. ^*^Statistically significant difference vs. isogenic shCTRL line; #Statistically significant difference vs. Ctrl shCTRL line; ^*^ and #*p* < 0.05; ##*p* < 0.01; ###*p* < 0.001. For each patient, one cell line per each reagent was used (9 modified cell lines in total).

We next asked whether downregulation of mutant HTT protein in shHTT2 iPSC lines would affect the pathways that were identified for HD and which were also affected in HD iPSC (Bowles and Jones, 2014; Szlachcic et al., 2015; Wiatr et al., 2017). Therefore, we examined β-catenin, phospho-β-catenin and phospho-ERK 1/2 by western blotting in HD YAC128 iPSC (Figure 4) and human HD109 and HD 71 iPSCs (Figure 5, Figure S5). In our previous study, we observed that more β-catenin protein is tagged for decay by Wnt-mediated phosphorylation at serines 33 and 37 in HD iPSCs (Szlachcic et al., 2015). The downregulation of mutant HTT did not affect this phenotype; however, the total β-catenin expression was increased (19 ± 6%, *p* = 0.0126) in mouse shHTT2-iPSC lines (Figures 4A,B). MAPK signaling is suppressed in HD YAC128 iPSCs, as indicated by the weaker ERK1/2 phosphorylation observed upon bFGF stimulation (Szlachcic et al., 2015). In the present study we observed an inconsistent response to HTT silencing (Figures 4C,D) showing no rescue (in isogenic lines derived from lines 1) or further decrease of ERK1/2 phosphorylation (in isogenic lines derived from line 2) after 30 min of bFGF stimulation (−87 ± 14%; shHTT2 vs. shCTRL Bonferroni *post-hoc* test *p* < 0.001).

In the case of Wnt in human cells (Figure 5) we observed a general increase in phospho-β-catenin in HD109 cells with HTT knockdown (+125 ± 44%; *p* < 0.05; shHTT2 vs shCTRL), while it was not changed in HD71 upon HTT knockdown. In HD109 and HD71 shCtrl lines total β-catenin level was increased as compared to Control shCtrl cells (+134 ± 40%, *p* < 0.05 and 174 ± 21%, *p* < 0.01, respectively). Silencing of HTT with shHTT2 in HD71 further increased the level of total β-catenin (39 ± 15%; *p* < 0.05; shHTT2 vs. shCTRL) but no significant increase was present in HD109 (42 ± 30%, not significant, shHTT2 vs. shCTRL). ERK1/2 phosphorylation was not affected in human HD109 and HD71 cells with shHTT vs shControl (Figure S6). In general we observed moderate effects of the HTT silencing on Wnt and Erk1/2 pathways in both mouse and human HD iPSC.

### 3.5. HTT silencing is able to reverse p53 deregulation in mouse isogenic iPSCs and NSCs

**Figure 6:**
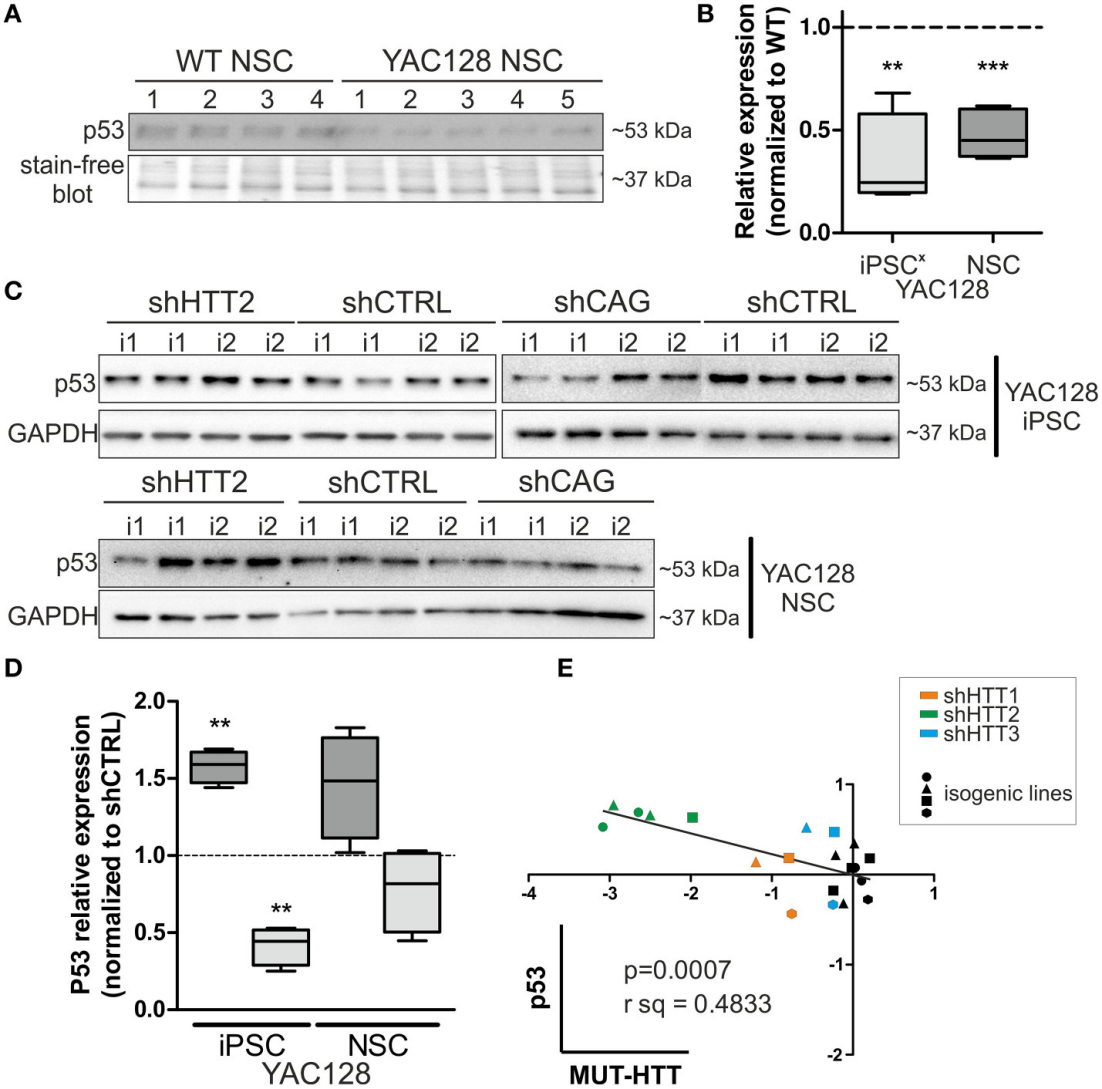
p53 protein levels in iPSC and NSC states are dependent on mutant HTT levels. **(A,B)** The decreased p53 level is maintained in HD YAC128 NSCs after differentiation of iPSCs, as assessed by western blotting (*N* = 4 for WT and *N* = 5 for YAC128). The data was normalized to total protein visualized on blots using Bio-rads stain-free technology. Data from YAC128 iPS cells (X) was adapted from our previous work (Szlachcic et al., 2015) (*N* = 6 for WT, *N* = 5 for YAC128). shHTT iPSC **(C)** and NSC **(D)** lines show reversal of the p53 phenotype, whereas shCAG reagents further decrease the p53 expression level. *N* = 4 for each reagent in both iPSC and NSC. **(E)** Pearson correlation reveals an inverse correlation of mutant HTT and p53 expression levels (*N* = 11 for HTT reagents and *N* = 9 for control shCTRL reagent). In NSCs, the correlation is not significant ^**^p < 0.01, ^***^p < 0.001. In Panel **(A)** and (**C**, iPSCs) blots were cropped; full-length blots are presented in Figure S8.

Along with our previous data showing decreased levels of p53 expression in YAC128 iPSCs and human juvenile HD iPSCs (Szlachcic et al., 2015), we found a similar decrease in p53 expression in NSCs originating from YAC128 iPSCs (−52 ± 7%, *p* = 0.0006) (Figures 6A,B). Subsequently, we investigated the YAC128 iPSCs and NSCs with shHTT and shCAG reagents vs isogenic shCTRL lines. In Figures 6C,D, we show that shHTT2 is able to rescue the decrease in p53 protein expression and to drive p53 expression well above the levels seen with the shCTRL reagent in both iPSCs and NSCs (56 ± 11%, *p* = 0.0003 and 44 ± 19%, *p* = 0.053, respectively). Interestingly, the shCAG reagent did not rescue p53 expression in iPSCs or NSCs and led to further decreases in the p53 levels (Figures 6C,D). The pattern of p53 deregulation was followed by similar deregulation of phospho-p53 (S15) (Figure S7). The correlation study on isogenic lines expressing all anti-HTT reagents (shHTT1-3) or shCTRL reagents revealed that the levels of mutant HTT and p53 were inversely correlated in iPSCs (Pearson *r* = −0.6952; *p* = 0.0007; Figure 6E).

**Figure 7:**
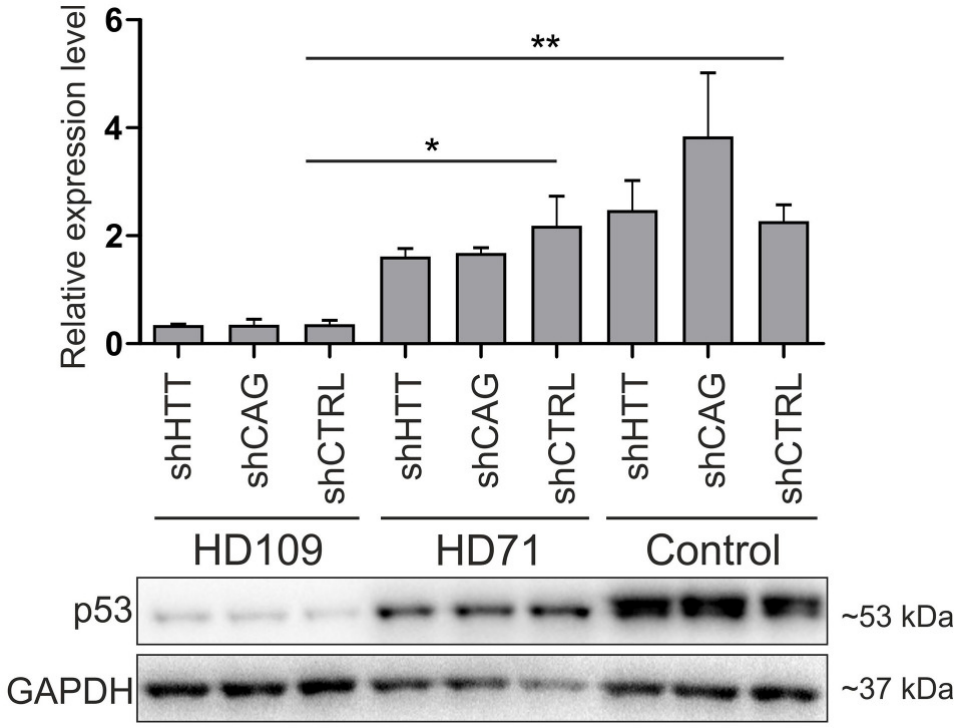
Non-allele selective silencing of HTT does not affect p53 expression level in human juvenile HD and control iPSCs. Western blot analysis of p53 expression in human HD109, HD71 and Control iPSCs with shHTT2, shCAG and shCTRL reagents. Although, in HD109 shCTRL line expression of p53 was significantly lower than in shCTRL HD71 (*p* = 0.0104) or Control line (*p* = 0.0045), shHTT reagent did not affect the phenotype in any of lines. ^*^p < 0.05; ^**^p < 0.01. For each patient, one cell line per each reagent was used (9 modified cell lines in total).

Although, p53 level was strongly decreased in human 109Q lines as compared to healthy control lines with shRNAs expression (−85 ± 15%, *p* < 0.001 between shCTRL lines), we found no effects of HTT silencing with shHTT2 reagent on the p53 expression level (Figure 7). In summary, we found the that the low level of p53 in mouse YAC128 iPSC and derived NSC was reversible after shHTT2, while the decreased level of p53 in human cells did not react to silencing.

## 4. Discussion

An experimental system in which the expression of a causative gene can be constantly depleted or eliminated should be considered for studying the pathogenesis of genetic neurodegenerative disorders and for therapeutic approaches. Therefore our aim was to generate HD iPSC lines with stable depletion of mutant HTT and to identify whether phenotypes characteristic for HD can be affected by HTT knockdown in HD iPSC. In addition, such approach allows for generation of cell lines with or without HTT silencing on the same, homogenous genetic background, i.e., isogenic lines. Use of isogenic lines reduces variability between compared cell lines that could mask discovery of relevant phenotypes.

**Figure 8:**
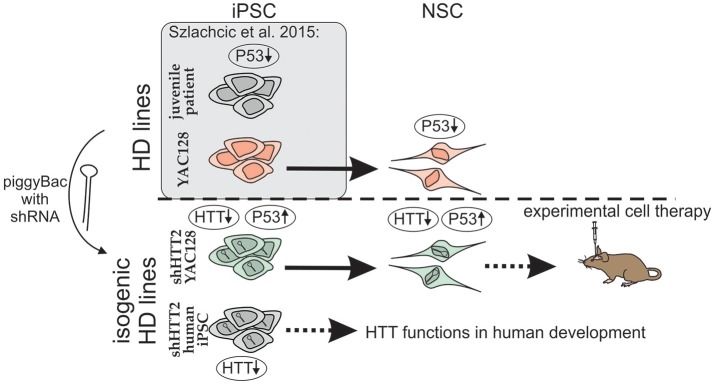
Schematic illustration of the isogenic experimental system. Isogenic iPSC lines with HTT silencing by shHTT2 were derived from previously described HD-YAC128 iPSCs and human HD109, HD71, and Control iPSCs. Knockdown of HTT in both YAC128 iPSCs and NSCs resulted in rescue of p53 downregulation phenotype. iPSCs with HTT knockdown can be used for experimental cell therapy (mouse YAC128 iPSCs) or research on huntingtin developmental functions (human iPSCs). Gene expression changes are indicated by upward (upregulation) or downward arrows (downregulation).

One of the suitable strategies for genetic correction of HD cells is the constant expression of shRNA and gene silencing. We used a piggyBac transposase system (Yusa et al., 2011) and anti-HTT shRNA in the mir-30 backbone (Paddison et al., 2004) which provides additional possibility for future excision of the reagent if desired. Using the strategy (Figure 8), we derived isogenic mouse YAC128 HD and human HD iPSCs with continuous expression of shRNA targeting HTT. In the case of mouse iPSC, we tested 3 reagents targeting human HTT shHTT1-3 and a reagent targeting CAG repeats in mutant HTT (shCAG). The most potent anti-HTT reagent, shHTT2, was continuously effective in both mouse iPSC and NSC lines with approximately 85 and 62% silencing of the mutant human protein, respectively; the silencing was similar between isogenic lines with different reagents. However, the shHTT2 reagent also reduced by approximately 53% expression of wild-type mouse huntingtin, which is expressed in YAC128 mice from 2 alleles. The shHTT2 reagent was also further used to generate shRNA expressing human HD109, HD71 and Control iPSCs. In human lines the reagent was silencing both mutant and normal allele with similarly high efficiency (over 80% silencing in HD71 and Control iPSCs). We also observed lower levels of both normal and mutant HTT in HD109 cells; however after knockdown with shHTT2 reagent the expression level of each allele reached similar levels in all lines, irrespectively of their expression levels in isogenic lines without silencing. The factor interfering with the HTT protein and transcript level may be a transcript retention in the nuclear foci demonstrated for HD cells which may result in prevention of the effective transport and translation of both mutant and normal transcripts (Urbanek et al., 2016). This may result in knock down of normal and mutant HTT in stem cells and insufficiency of HTT during development in juvenile patients. Low level of normal huntingtin may also indicate that its silencing via non-allele specific strategies might be detrimental in the case of juvenile HD.

The action of the shCAG reagent whose design was based on published allele-selective reagent revealed a dependency on cell type. In mouse iPSCs, the reagent increased mutant HTT expression, in mouse NSCs it induced a 40% silencing effect, while in human iPS cells it remained ineffective. In previous studies, the shCAG reagent was very effective at silencing HTT in fibroblasts (Fiszer et al., 2013); the observed differences in shCAG activity might result from differences in processing of the reagent in different cell types (Meijer et al., 2014; Tan et al., 2014). The described phenomena may depend on differences in the construct properties and the reagent delivery, e.g., our piggyBac construct vs. a lentiviral construct (Fiszer et al., 2013). Therefore, the shCAG reagent might be effective and should be examined in terminally differentiated neurons. Moreover, our results indicate the necessity of tailoring the therapeutic shRNA and delivery systems for disease-specific cell types, e.g., terminally differentiated neurons or astrocytes in the case of HD. In addition, the shCAG reagent demonstrates a new feature of our system namely as a reagent testing pipeline where the effects of reagents can be more precisely tested in consecutive cellular stages.

We have previously reported that YAC128 iPSCs exhibit phenotypes of early HD and share these phenotypes with human iPSCs from juvenile HD patients (Szlachcic et al., 2015). Among the changes, we identified decreased MAPK1 activation and p53 levels and increased β-catenin-p(33/37) levels. Using the established system for continuous silencing we have assessed whether the HD phenotypes demonstrated a dependency on the level of mutant HTT. In the case of the MAPK pathway and iPSCs, we observed minor changes in ERK1/2 activation depending on the iPSC line. In the case of mouse iPSC and Wnt pathway, the total β-catenin level had a slight dependency on the HTT level. Also in HD71 we have seen a greater total HTT level which may indicate recovery of Wnt signaling. In the case of HD109 we have demonstrated even higher phosphorylation of β-catenin, which may indicate an adverse effect on Wnt. We conclude that more human iPSC more HD iPS cell lines with mutant allele selective silencing originating from several patients are needed to investigate a fine relation of HTT expression and the pathways in iPSC. The differences in the phenotypes between HD109 and HD71 iPSC may be also attributed to number of CAG repeats in HTT but we can also not exclude the effect of the genetic background.

A large difference in p53 expression was found in the isogenic mouse iPSC and NSC. First, we demonstrated that p53 downregulation continued through to the NSC stage and the difference was similar to one previously seen by us for iPSC (Szlachcic et al., 2015). Moreover, shRNA isogenic cell lines at the iPSC and NSC stages demonstrated a clear dependency of p53 level on the mutant HTT level, although the effect was less prominent in NSCs. Interestingly, we observed recovery of decreased p53 expression with the shHTT2 reagent and a lack of recovery and a further decline in p53 expression with shCAG in both cell types. In the case of human HD109 iPS cells expressing the shHTT2 we did not observe the recovery of p53 level. Previously, p53 was shown to be involved in HD pathogenesis, with total levels in the brain increasing with HD severity and particularly being upregulated in late HD stages, in grade 3 and 4 patients (Bae et al., 2005). Although, p53 protein expression was upregulated in YAC128 mice, this upregulation was not observed in primary neuronal culture from E16.5 YAC128 mice unless cells were treated with the p53 activator camptothecin (Ehrnhoefer et al., 2013). Moreover, p53 phosphorylation and activation of the ATM DNA-damage-response pathway are downregulated in HD iPSCs (Tidball et al., 2015). Our results, together with the published data, may indicate that the gradient of p53 expression changes from downregulation in early HD stages (stem and NSCs) to upregulation in the adult brain during neurodegeneration. In general, it is well established that fine regulation of p53 expression in early developmental stages is essential to maintain the necessary balance between stem cell self-renewal and differentiation (Yang et al., 2014). In addition, high p53 expression levels may lead to terminal differentiation and growth arrest (Mendrysa et al., 2011). Decreased p53 levels in neurodegenerative diseases may lead to excessive NSC activation and insufficient differentiation potential, similar to what is observed in fragile X syndrome model (Li et al., 2016). NSCs from HD mouse models also show enhanced late-stage self-renewal, delayed cell cycle exit and impaired differentiation into striatal medium spiny neurons subtype (Molero et al., 2009; Molina-Calavita et al., 2014). Therefore, p53 may be a valid early therapeutic target in neurodegenerative diseases. Similar to p53 the Wnt is one of the major signaling pathways during development, it remains active in adult brain and is implicated in brain diseases (Noelanders and Vleminckx, 2017). In addition, the clear dependency of p53 on HTT expression in mouse cells and its downregulation in HD109 iPSCs implicates p53 in potential therapy in juvenile HD.

Sustained silencing of HTT via shRNA in iPSCs and NSCs, together with the observed p53 phenotype reversal, supports the idea of combining shRNA and autologous cell therapy. In therapeutic applications for HD, simple excision of the mutant allele via homologous recombination (An et al., 2012) or genome editing using, e.g., a CRISPR-Cas9 system, may be insufficient and could be accompanied by stable shRNA expression. An important reason for combining both approaches or even for selecting shRNA for cell therapy is evident from several demonstrations of neurodegeneration (including HTT aggregate formation) of healthy cells grafted into a brain undergoing neurodegeneration (Cicchetti et al., 2009, 2011, 2014; Jeon et al., 2016). One of the underlying mechanisms is the shuttling of HTT mRNA or HTT protein aggregates between cells, thus allowing them to spreading through the graft in a prion-like manner (Brundin et al., 2010; Herrera and Outeiro, 2012; Costanzo et al., 2013; Pecho-Vrieseling et al., 2014; Jeon et al., 2016; Zhang et al., 2016). Moreover, it is known that cells, including neurons and glia, can mediate exosomal and non-exosomal transfer of both proteins and RNAs (Vlassov et al., 2012; Frühbeis et al., 2013), including miRNAs (Wang et al., 2010; Hu et al., 2012) and synthetic mature shRNAs (Olson et al., 2012). Therefore, shRNA from grafted cells, if transferred via exosomes, may confer a therapeutic effect on host cells. Taken together, sustained expression of an HTT-silencing agent that also reverses the molecular phenotype has the capacity to protect grafts from non-cell-autonomous degeneration caused by surrounding HD-affected host cells.

To our knowledge, our human HD and Control iPSC lines with integrated shHTT2 are the first human iPSCs with stable huntingtin knock-down, and they can be helpful for *in vitro* research on huntingtin functions in human development.

## Author contributions

WS and MacF conceived, designed, performed the experiments, and analyzed the data. KW performed ERK assay and consecutive western blotting analyses. KW and MT cultured and analyzed the phenotype of NSC. MacF planned and executed the live animal experiments (mouse brain injections). MarF critically revised the article. WS and MacF wrote the paper. MacF was responsible for concept and obtaining funding.

### Conflict of interest statement

The authors declare that the research was conducted in the absence of any commercial or financial relationships that could be construed as a potential conflict of interest.

## Abbreviations

HD: Huntington disease
iPSCs: induced pluripotent stem cells
NSC: neural stem cells
HTT: huntingtin.

## References

[B1] An M. C. Zhang N. Scott G. Montoro D. Wittkop T. Mooney S.. (2012). Genetic correction of Huntington's disease phenotypes in induced pluripotent stem cells. Cell Stem Cell 11, 253–263. 10.1016/j.stem.2012.04.026 22748967 PMC3608272

[B2] Bae B.-I. Xu H. Igarashi S. Fujimuro M. Agrawal N. Taya Y.. (2005). p53 mediates cellular dysfunction and behavioral abnormalities in Huntington's disease. Neuron 47, 29–41. 10.1016/j.neuron.2005.06.005 15996546

[B3] Bates G. P. Dorsey R. Gusella J. F. Hayden M. R. Kay C. Leavitt B. R.. (2015). Huntington disease. Nat. Rev. Dis. Primers 1:15005. 10.1038/nrdp.2015.5 27188817

[B4] Bowles K. R. Jones L. (2014). Kinase signalling in Huntington's disease. J. Huntingtons Dis. 3, 89–123. 10.3233/JHD-140106 25062854

[B5] Brundin P. Melki R. Kopito R. (2010). Prion-like transmission of protein aggregates in neurodegenerative diseases. Nat. Rev. Mol. Cell Biol. 11, 301–307. 10.1038/nrm2873 20308987 PMC2892479

[B6] Cicchetti F. Lacroix S. Cisbani G. Vallières N. Saint-Pierre M. St-Amour I.. (2014). Mutant huntingtin is present in neuronal grafts in Huntington disease patients. Ann. Neurol. 76, 31–42. 10.1002/ana.24174 24798518

[B7] Cicchetti F. Saporta S. Hauser R. A. Parent M. Saint-Pierre M. Sanberg P. R.. (2009). Neural transplants in patients with Huntington's disease undergo disease-like neuronal degeneration. Proc. Natl. Acad. Sci. U.S.A. 106, 12483–12488. 10.1073/pnas.0904239106 19620721 PMC2713393

[B8] Cicchetti F. Soulet D. Freeman T. B. (2011). Neuronal degeneration in striatal transplants and Huntington's disease: potential mechanisms and clinical implications. Brain 134(Pt 3), 641–652. 10.1093/brain/awq328 21278084

[B9] Costanzo M. Abounit S. Marzo L. Danckaert A. Chamoun Z. Roux P.. (2013). Transfer of polyglutamine aggregates in neuronal cells occurs in tunneling nanotubes. J. Cell. Sci. 126(Pt 16), 3678–3685. 10.1242/jcs.126086 23781027

[B10] Duyao M. P. Auerbach A. B. Ryan A. Persichetti F. Barnes G. T. McNeil S. M.. (1995). Inactivation of the mouse Huntington's disease gene homolog Hdh. Science 269, 407–410. 7618107 10.1126/science.7618107

[B11] Ebert A. D. Shelley B. C. Hurley A. M. Onorati M. Castiglioni V. Patitucci T. N.. (2013). EZ spheres: a stable and expandable culture system for the generation of pre-rosette multipotent stem cells from human ESCs and iPSCs. Stem Cell Res. 10, 417–427. 10.1016/j.scr.2013.01.009 23474892 PMC3786426

[B12] Ehrnhoefer D. E. Skotte N. H. Ladha S. Nguyen Y. T. N. Qiu X. Deng Y.. (2013). p53 increases caspase-6 expression and activation in muscle tissue expressing mutant huntingtin. Hum. Mol. Genet. 23, 717–729. 10.1093/hmg/ddt458 24070868

[B13] Fiszer A. Olejniczak M. Galka-Marciniak P. Mykowska A. Krzyzosiak W. J. (2013). Self-duplexing CUG repeats selectively inhibit mutant huntingtin expression. Nucleic Acids Res. 41, 10426–10437. 10.1093/nar/gkt825 24038471 PMC3905887

[B14] Frühbeis C. Fröhlich D. Kuo W. P. Krämer-Albers E.-M. (2013). Extracellular vesicles as mediators of neuron-glia communication. Front. Cell. Neurosci. 7:182. 10.3389/fncel.2013.00182 24194697 PMC3812991

[B15] Halfter W. Dong S. Yip Y.-P. Willem M. Mayer U. (2002). A critical function of the pial basement membrane in cortical histogenesis. J. Neurosci. 22, 6029–6040. 12122064 10.1523/JNEUROSCI.22-14-06029.2002 PMC6757907

[B16] Herrera F. Outeiro T. F. (2012). α-Synuclein modifies huntingtin aggregation in living cells. FEBS Lett. 586, 7–12. 10.1016/j.febslet.2011.11.019 22119730

[B17] Hockfield S. McKay R. D. (1985). Identification of major cell classes in the developing mammalian nervous system. J. Neurosci. 5, 3310–3328. 4078630 10.1523/JNEUROSCI.05-12-03310.1985 PMC6565218

[B18] Hu G. Drescher K. M. Chen X.-M. (2012). Exosomal miRNAs: biological properties and therapeutic potential. Front. Genet. 3:56. 10.3389/fgene.2012.00056 22529849 PMC3330238

[B19] Jeon I. Cicchetti F. Cisbani G. Lee S. Li E. Bae J.. (2016). Human-to-mouse prion-like propagation of mutant huntingtin protein. Acta Neuropathol. 132, 577–592. 10.1007/s00401-016-1582-9 27221146 PMC5023734

[B20] Ko J. Ou S. Patterson P. H. (2001). New anti-huntingtin monoclonal antibodies: implications for huntingtin conformation and its binding proteins. Brain Res. Bull. 56, 319–329. 10.1016/S0361-9230(01)00599-8 11719267

[B21] Kordasiewicz H. B. Stanek L. M. Wancewicz E. V. Mazur C. McAlonis M. M. Pytel K. A.. (2012). Sustained therapeutic reversal of Huntington's disease by transient repression of huntingtin synthesis. Neuron 74, 1031–1044. 10.1016/j.neuron.2012.05.009 22726834 PMC3383626

[B22] Li Y. Stockton M. E. Bhuiyan I. Eisinger B. E. Gao Y. Miller J. L.. (2016). MDM2 inhibition rescues neurogenic and cognitive deficits in a mouse model of fragile X syndrome. Sci. Transl. Med. 8:336ra61. 10.1126/scitranslmed.aad9370 27122614 PMC4995450

[B23] Macdonald D. Tessari M. Boogaard I. Smith M. Pulli K. Szynol A.. (2014). Quantification assays for total and polyglutamine-expanded huntingtin proteins. PLoS ONE 9:e96854. 10.1371/journal.pone.0096854 24816435 PMC4016121

[B24] Martí E. (2016). RNA toxicity induced by expanded CAG repeats in Huntington's disease. Brain Pathol. 26, 779–786. 10.1111/bpa.12427 27529325 PMC8029335

[B25] Mattis V. B. Svendsen C. N. (2015). Modeling Huntington's disease with patient-derived neurons. Brain Res. 1656, 76–87. 10.1016/j.brainres.2015.10.001 26459990

[B26] McKinstry S. U. Karadeniz Y. B. Worthington A. K. Hayrapetyan V. Y. Ozlu M. I. Serafin-Molina K.. (2014). Huntingtin is required for normal excitatory synapse development in cortical and striatal circuits. J. Neurosci. 34, 9455–9472. 10.1523/JNEUROSCI.4699-13.2014 25009276 PMC4087216

[B27] Meijer H. A. Smith E. M. Bushell M. (2014). Regulation of miRNA strand selection: follow the leader? Biochem. Soc. Trans. 42, 1135–1140. 10.1042/BST20140142 25110015

[B28] Mendrysa S. M. Ghassemifar S. Malek R. (2011). p53 in the CNS: perspectives on development, stem cells, and cancer. Genes Cancer 2, 431–442. 10.1177/1947601911409736 21779511 PMC3135640

[B29] Miniarikova J. Zanella I. Huseinovic A. van der Zon T. Hanemaaijer E. Martier R.. (2016). Design, Characterization, and lead selection of therapeutic miRNAs targeting huntingtin for development of gene therapy for Huntington's disease. Mol. Ther. Nucleic Acids 5:e297. 10.1038/mtna.2016.7 27003755 PMC5014463

[B30] Molero A. E. Arteaga-Bracho E. E. Chen C. H. Gulinello M. Winchester M. L. Pichamoorthy N.. (2016). Selective expression of mutant huntingtin during development recapitulates characteristic features of Huntington's disease. Proc. Natl. Acad. Sci. U.S.A. 113, 5736–5741. 10.1073/pnas.1603871113 27140644 PMC4878495

[B31] Molero A. E. Gokhan S. Gonzalez S. Feig J. L. Alexandre L. C. Mehler M. F. (2009). Impairment of developmental stem cell-mediated striatal neurogenesis and pluripotency genes in a knock-in model of Huntington's disease. Proc. Natl. Acad. Sci. U.S.A. 106, 21900–21905. 10.1073/pnas.0912171106 19955426 PMC2799796

[B32] Molina-Calavita M. Barnat M. Elias S. Aparicio E. Piel M. Humbert S. (2014). Mutant huntingtin affects cortical progenitor cell division and development of the mouse neocortex. J. Neurosci. 34, 10034–10040. 10.1523/JNEUROSCI.0715-14.2014 25057205 PMC6608303

[B33] Nasir J. Floresco S. B. O'Kusky J. R. Diewert V. M. Richman J. M. Zeisler J.. (1995). Targeted disruption of the Huntington's disease gene results in embryonic lethality and behavioral and morphological changes in heterozygotes. Cell 81, 811–823. 10.1016/0092-8674(95)90542-1 7774020

[B34] Noelanders R. Vleminckx K. (2017). How Wnt signaling builds the brain: bridging development and disease. Neuroscientist 23, 314–329. 10.1177/1073858416667270 27624848

[B35] Olson A. Sheth N. Lee J. S. Hannon G. Sachidanandam R. (2006). RNAi Codex: a portal/database for short-hairpin RNA (shRNA) gene-silencing constructs. Nucleic Acids Res. 34(Database issue), D153–D157. 10.1093/nar/gkj051 16381835 PMC1347414

[B36] Olson S. D. Kambal A. Pollock K. Mitchell G.-M. Stewart H. Kalomoiris S.. (2012). Examination of mesenchymal stem cell-mediated RNAi transfer to Huntington's disease affected neuronal cells for reduction of huntingtin. Mol. Cell. Neurosci. 49, 271–281. 10.1016/j.mcn.2011.12.001 22198539 PMC3784251

[B37] Paddison P. J. Cleary M. Silva J. M. Chang K. Sheth N. Sachidanandam R.. (2004). Cloning of short hairpin RNAs for gene knockdown in mammalian cells. Nat. Methods 1, 163–167. 10.1038/nmeth1104-163 16144086

[B38] Pecho-Vrieseling E. Rieker C. Fuchs S. Bleckmann D. Esposito M. S. Botta P.. (2014). Transneuronal propagation of mutant huntingtin contributes to non-cell autonomous pathology in neurons. Nat. Neurosci. 17, 1064–1072. 10.1038/nn.3761 25017010

[B39] Quigley J. (2017). Juvenile Huntington's disease: diagnostic and treatment considerations for the psychiatrist. Curr. Psychiatry Rep. 19:9. 10.1007/s11920-017-0759-9 28168595

[B40] Rué L. Banez-Coronel M. Creus-Muncunill J. Giralt A. Alcalá-Vida R. Mentxaka G.. (2016). Targeting CAG repeat RNAs reduces Huntington's disease phenotype independently of huntingtin levels. J. Clin. Invest. 126, 4319–4330. 10.1172/JCI83185 27721240 PMC5096913

[B41] Shaner N. C. Lin M. Z. McKeown M. R. Steinbach P. A. Hazelwood K. L. Davidson M. W.. (2008). Improving the photostability of bright monomeric orange and red fluorescent proteins. Nat. Methods 5, 545–551. 10.1038/nmeth.1209 18454154 PMC2853173

[B42] Slow E. J. van Raamsdonk J. Rogers D. Coleman S. H. Graham R. K. Deng Y.. (2003). Selective striatal neuronal loss in a YAC128 mouse model of Huntington disease. Hum. Mol. Genet. 12, 1555–1567. 10.1093/hmg/ddg169 12812983

[B43] Squitieri F. Frati L. Ciarmiello A. Lastoria S. Quarrell O. (2006). Juvenile Huntington's disease: does a dosage-effect pathogenic mechanism differ from the classical adult disease? Mech. Ageing Dev. 127, 208–212. 10.1016/j.mad.2005.09.012 16274727

[B44] Svendsen C. N. ter Borg M. G. Armstrong R. J. Rosser A. E. Chandran S. Ostenfeld T.. (1998). A new method for the rapid and long term growth of human neural precursor cells. J. Neurosci. Methods 85, 141–152. 10.1016/S0165-0270(98)00126-5 9874150

[B45] Szlachcic W. J. Switonski P. M. Krzyzosiak W. J. Figlerowicz M. Figiel M. (2015). Huntington disease iPSCs show early molecular changes in intracellular signaling, the expression of oxidative stress proteins and the p53 pathway. Dis. Model Mech. 8, 1047–1057. 10.1242/dmm.019406 26092128 PMC4582098

[B46] Tabrizi S. J. Scahill R. I. Owen G. Durr A. Leavitt B. R. Roos R. A.. (2013). Predictors of phenotypic progression and disease onset in premanifest and early-stage Huntington's disease in the TRACK-HD study: analysis of 36-month observational data. Lancet Neurol. 12, 637–649. 10.1016/S1474-4422(13)70088-7 23664844

[B47] Tan G. C. Chan E. Molnar A. Sarkar R. Alexieva D. Isa I. M.. (2014). 5' isomiR variation is of functional and evolutionary importance. Nucleic Acids Res. 42, 9424–9435. 10.1093/nar/gku656 25056318 PMC4132760

[B48] The-Huntington's-Disease-Collaborative-Research-Group (1993). A novel gene containing a trinucleotide repeat that is expanded and unstable on Huntington's disease chromosomes. Cell 72, 971–983. 8458085 10.1016/0092-8674(93)90585-e

[B49] Tidball A. M. Bryan M. R. Uhouse M. A. Kumar K. K. Aboud A. A. Feist J. E.. (2015). A novel manganese-dependent ATM-p53 signaling pathway is selectively impaired in patient-based neuroprogenitor and murine striatal models of Huntington's disease. Hum. Mol. Genet. 24, 1929–1944. 10.1093/hmg/ddu609 25489053 PMC4355025

[B50] Urbanek M. O. Jazurek M. Switonski P. M. Figura G. Krzyzosiak W. J. (2016). Nuclear speckles are detention centers for transcripts containing expanded cag repeats. Biochim. Biophys. Acta 1862, 1513–1520. 10.1016/j.bbadis.2016.05.015 27239700

[B51] Vlassov A. V. Magdaleno S. Setterquist R. Conrad R. (2012). Exosomes: current knowledge of their composition, biological functions, and diagnostic and therapeutic potentials. Biochim. Biophys. Acta 1820, 940–948. 10.1016/j.bbagen.2012.03.017 22503788

[B52] Wang K. Zhang S. Weber J. Baxter D. Galas D. J. (2010). Export of microRNAs and microRNA-protective protein by mammalian cells. Nucleic Acids Res. 38, 7248–7259. 10.1093/nar/gkq601 20615901 PMC2978372

[B53] Wiatr K. Szlachcic W. J. Trzeciak M. Figlerowicz M. Figiel M. (2017). Huntington disease as a neurodevelopmental disorder and early signs of the disease in stem cells. Mol. Neurobiol. [Epub ahead of print]. 10.1007/s12035-017-0477-7 28497201 PMC5842500

[B54] Yang B. Treweek J. B. Kulkarni R. P. Deverman B. E. Chen C.-K. Lubeck E.. (2014). Single-cell phenotyping within transparent intact tissue through whole-body clearing. Cell 158, 945–958. 10.1016/j.cell.2014.07.017 25088144 PMC4153367

[B55] Yusa K. Rad R. Takeda J. Bradley A. (2009). Generation of transgene-free induced pluripotent mouse stem cells by the piggyBac transposon. Nat. Methods 6, 363–369. 10.1038/nmeth.1323 19337237 PMC2677165

[B56] Yusa K. Zhou L. Li M. A. Bradley A. Craig N. L. (2011). A hyperactive piggyBac transposase for mammalian applications. Proc. Natl. Acad. Sci. U.S.A. 108, 1531–1536. 10.1073/pnas.1008322108 21205896 PMC3029773

[B57] Zeitlin S. Liu J. P. Chapman D. L. Papaioannou V. E. Efstratiadis A. (1995). Increased apoptosis and early embryonic lethality in mice nullizygous for the Huntington's disease gene homologue. Nat. Genet. 11, 155–163. 10.1038/ng1095-155 7550343

[B58] Zhang N. Bailus B. J. Ring K. L. Ellerby L. M. (2015). iPSC-based drug screening for Huntington's disease. Brain Res. 1638(Pt A), 42–56. 10.1016/j.brainres.2015.09.020 26428226 PMC4814369

[B59] Zhang X. Abels E. R. Redzic J. S. Margulis J. Finkbeiner S. Breakefield X. O. (2016). Potential transfer of polyglutamine and CAG-repeat RNA in extracellular vesicles in Huntington's disease: background and evaluation in cell culture. Cell. Mol. Neurobiol. 36, 459–470. 10.1007/s10571-016-0350-7 26951563 PMC5844350

