# Corrigendum: The Generation of Mouse and Human Huntington Disease iPS Cells Suitable for *In vitro* Studies on Huntingtin Function

**DOI:** 10.3389/fnmol.2017.00312

**Published:** 2017-09-28

**Authors:** Wojciech J. Szlachcic, Kalina Wiatr, Marta Trzeciak, Marek Figlerowicz, Maciej Figiel

**Affiliations:** ^1^Department of Molecular Neurobiology, Institute of Bioorganic Chemistry, Polish Academy of Sciences, Poznań, Poland; ^2^Department of Molecular and Systems Biology, Institute of Bioorganic Chemistry, Polish Academy of Sciences, Poznań, Poland

**Keywords:** Huntington disease, iPS cells, NS cells, YAC128, shRNA, huntingtin, p53, juvenile HD

In the original article, in the Acknowledgments section there was a missing sentence with acknowledgments for Pawel M. Switonski. The authors apologize for this error and state that this does not change the scientific conclusions of the article in any way.

The following sentence was missing from the Acknowledgments section: “We kindly thank Pawel M. Switonski for his contribution to designing the psiOrange constructs.” Therefore, the Acknowledgments section should have read as follows.

## Conflict of interest statement

The authors declare that the research was conducted in the absence of any commercial or financial relationships that could be construed as a potential conflict of interest.

