# Supplementary material for: The Generation of Mouse and Human Huntington Disease iPS Cells Suitable for In vitro Studies on Huntingtin Function
Source: Front Mol Neurosci. 2017 Aug 8;10:253. doi: 10.3389/fnmol.2017.00253 (PMC5550714; doi:10.3389/fnmol.2017.00253)
Supplement: Supplementary file 1 [file Image1.PDF]

New mouse and human Huntington disease iPS cells with stable expression of anti-huntingtin shRNAs suitable for research on HD pathogenesis and HTT function

SUPPLEMENTARY DATA

Supplementary Methods

Cell culture

Adherent NS (aNSC) culture was initiated by dissociation of spheres with a single round of chopping followed by incubation with TrypLE. Spheres were dissociated into single cells and plated into gelatin-coated vessels at a density of 30,000 cells/cm^2. The aNSC medium consisted of DMEM/F12 (1:1) with 5 mM HEPES, additional glucose (6 mg/mL in total), 1x Glutamax, 1x N2 supplement, 50 μg/mL apo-transferrin (Sigma-Aldrich), 50 μg/mL BSA, 5 μg/mL heparin, 20 ng/mL bFGF and 20 ng/mL EGF. The N2 supplement consisted of 5 μg/mL insulin, 30 nM sodium selenite, 51 nM triiodo-L-thyronine (T3), 6 ng/mL progesterone, and 16 μg/mL putrescine (all Sigma-Aldrich). For the initial passages, cells were passaged 1:2 to 1:3 every 4-6 days; for later passages ($>$5), confluence was reached between 2-4 days when cells were passaged 1:3 to 1:4. The aNSCs were passaged with TrypLE, and culture medium was gently replaced every other day. For neuronal differentiation, cells were transferred into poly-DL-ornithine- and poly-D-lysine-coated wells in NSC medium without bFGF, EGF or heparin. For astrocyte differentiation, dishes were coated with gelatin and NSC medium without growth factors and was supplemented with 1% FBS. Neurons and astrocytes were immunostained after 14 days.

Striatal transplantation

NSC spheres were prepared for transplantation by priming cultures for 3 days without EGF53. Spheres were transferred to an animal facility in NSC medium on ice, where they were dissociated into single cells using chemical dissociation (NeuroCult Chemical Dissociation Kit; StemCell Technologies, Vancouver, Canada) and passed through a mesh filter (BD cell strainer, BD Biosciences, San Jose, CA). Then, 1x10^5 cells suspended in 2 μl of NS medium (w/o EGF) were stereotactically injected (Angle Two^{TM} Stereotaxic Instrument, Leica) using a Hamilton syringe with a 35G needle into the striatum at coordinates of AP +0.5, ML+1.8, DV-3.5. Mice were sacrificed at 8 weeks post-transplantation. The mOrange2 signal from transplanted cells was visualized after passive clearing of the brain using the PACT method (Yang et al., 2014).

qPCR analysis of HTT expression

Total mRNA was isolated from human HD iPSCs using RNAzol RT (Molecular Research Center, Cincinnati, OH) according to manufacturer's protocol. Reverse transcription was performed using Maxima H Minus Reverse Transcriptase (Thermo Fisher) (200U per reaction) on 2 μg of RNA in 20 μl of total reaction according to the manufacturer's protocol. For priming a mixture of random hexamers (25 pmol) and oligo(dT)18 (25 pmol) was used. Additionaly RiboLock RNase inhibitor was added

to the reaction mix (20U). Before adding the enzyme and the inhibitor, templates were denatured in 65°C for 5 minutes; after mixing all reaction reagents reaction was incubated for 10 min at 25°C followed by 15 min at 50°C. Resulting cDNA was further 10 times diluted with nuclease-free water and stored in -20°C. qPCR was performed using 5x HOT FIREPol EvaGreen qPCR Mix Plus (Solis BioDyne, Tartu, Estonia) on 1 μl of cDNA in 10 μl of total reaction volume. The reaction mix included 250 nM primers. Primers used are: exon1-Forward 5'-GAGCCGCTGCACCGAC-3', exon2-Reverse 5'-CTGACAGACTGTGCCACTATGTTT-3 (both adapted from (Sathasivam et al., 2013), exon52-Forward 5'-TCTCACGCCATTGCTAAGGA-3', exon53-Reverse 5'-TGACCATCCAAGCTTCCACA-3, PGK1-Forward 5'-CTGTGGCTTCTGGCATACCT-3', PGK1-Reverse 5'-CGAGTGACAGCCTCAGCATA-3'. Thermocycling parameters were as follows: 15 min of initial denaturation at 95°C and 45 two-step cycles with 20 s denaturation at 95°C, 20 s annealing at 60°C and 20 s elongation at 72°C. The reactions were run on CFX96 instrument (Bio-Rad). Specificity of reaction for each primer pair was confirmed by Melting curve analysis and agarose gel electrophoresis. RT- and no template controls were included in analysis. Data was obtained and analyzed using CFX Manager 3.1 (BioRad). Cq values were determined in software using regression model and were exported to Excel for further analyses. Relative expression level was calculated using dCt method with PGK1 used as a reference gene.

REFERENCES

Fiszer, A., Olejniczak, M., Galka-Marciniak, P., Mykowska, A., and Krzyzosiak, W. J. (2013). Self-duplexing CUG repeats selectively inhibit mutant huntingtin expression. *Nucleic Acids Res.* 41, 10426–10437. doi:10.1093/nar/gkt825

Sathasivam, K., Neueder, A., Gipson, T. A., Landles, C., Benjamin, A. C., Bondulich, M. K., et al. (2013). Aberrant splicing of HTT generates the pathogenic exon 1 protein in Huntington disease. *Proceedings of the National Academy of Sciences of the United States of America* 110, 2366–2370. doi:10.1073/pnas.1221891110

Yang, B., Treweek, J. B., Kulkarni, R. P., Deverman, B. E., Chen, C.-K., Lubeck, E., et al. (2014). Single-cell phenotyping within transparent intact tissue through whole-body clearing. *Cell* 158, 945–958. doi:10.1016/j.cell.2014.07.017

Table S1. Sequences of used shRNA guide strands

Name	Sequence	Target	Source
shHTT1	5'-UUUCGUAAACAAGUCAGCAGCC-3'	human HTT exon 67	HP_124353; RNAi Codex database
shHTT2	5'-UAUAGCCUCUAUAUAUUCUGGU-3'	human HTT exon 22/23	HP_7232; RNAi Codex database
shHTT3	5'-AAUACAAAGCCAAUAAACACCU-3'	human HTT exon 33/34	HP_220452; RNAi Codex database
shCAG	5'-GCUGCUGC**A**GCUGCUGCUGCUU-3'	CAG repeats in HTT **(one mismatch)**	A2_R reagent; (Fiszer et al., 2013)
shCTRL	5'-UUGAAGUUCACCUUGAUGCCGU-3'	GFP	this research

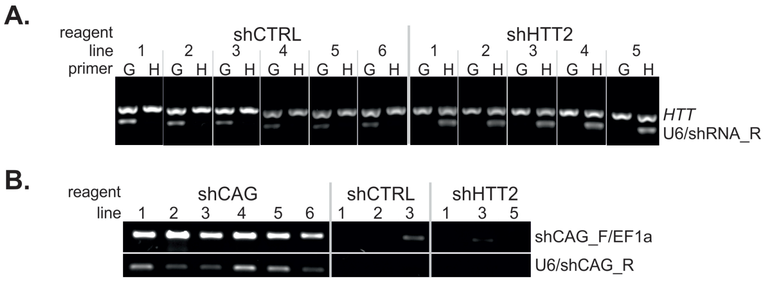

Figure S1: Genotyping analysis results on mouse iPSC lines to determine proper integration of shRNA transposons. In (A), multiplex PCR was performed with upper bands being the amplification products of human HTT, used as an internal reaction control, while lower bands are shHTT (H) or shCTRL (G) construct-specific products. In (B), we used two pairs of primers specific for 3' (lower panel) or 5' (upper panel) parts of shCAG.

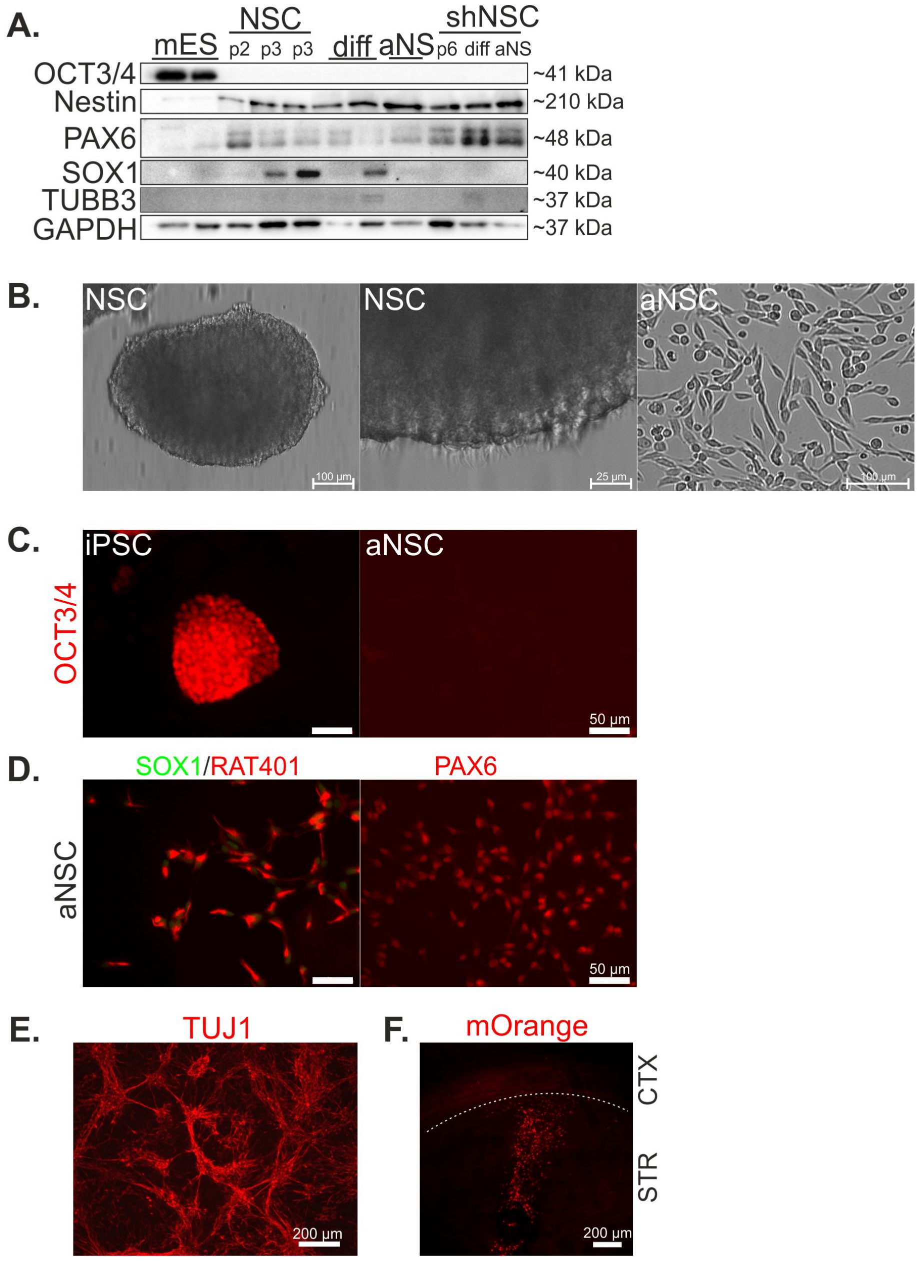

Figure S2: Characteristics of NSCs. (A) Western blot analysis of NSCs. Upon differentiation of iPSCs into floating NSC spheres, OCT3/4 expression is lost; this is maintained upon further passages, transition to adherent culture (aNSC) and further differentiation (diff). The expression of the general NSC markers, nestin and PAX6, is acquired and maintained upon passaging. In contrast, SOX1 expression peaks transiently in lower passages and is lost upon continuous culture. The neural marker TUBB3 is not present until after short-term differentiation of NSCs. (B) Morphology of NSCs. NSC spheres develop characteristic cilia (middle panel). After transfer of floating NSCs into adherent aNSCs, the cells acquire bipolar morphology. (C) Immunostaining of iPSCs and aNSCs for OCT3/4 confirms loss of its expression in the latter. (D) Nestin (RAT401 antibody) and PAX6 are expressed in the aNSC state, while SOX1 expression is not detected. (E) NSCs can be differentiated towards TUJ1-positive neurons. (F) Maximum-intensity projection of mOrange2 fluorescence in a whole graft within a CLARITY/PACT-cleared mouse brain, acquired via confocal microscopy. mOrange2-positive cells survive for at least 8 weeks after NSCs are transplanted into the mouse striatum.

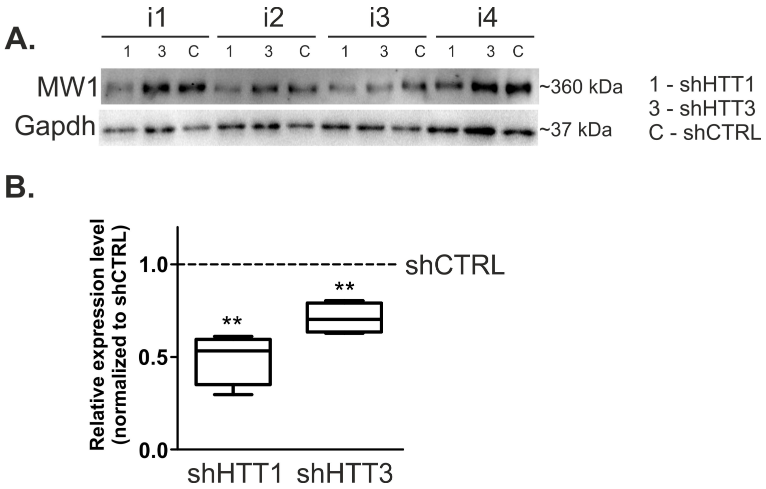

Figure S3: Efficiency of shHTT1 and shHTT3 reagents in mouse HD iPSCs. (A, B) Western blot analysis of mutant huntingtin expression level using polyQ-specific antibody MW1 as compared to shCTRL lines. * $p<0.05$, *** $p<0.001$; i1-i4 – isogenic lines derived from separate parental lines 1 to 4.

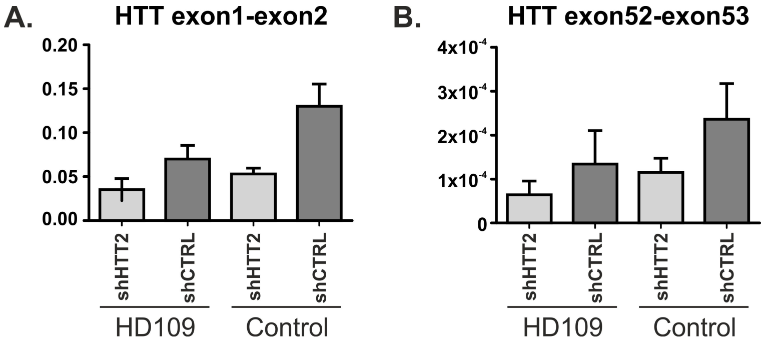

Figure S4: qPCR analysis of total HTT expression in HD109 and Control lines with shHTT2 vs shCTRL lines. Amplification of 5' region (exon1/exon2 junction) and of more terminal region (exon52/53 junction) HTT transcripts reveal similar decrease upon shHTT2 reagent-mediated silencing. Note lower levels of total HTT mRNAs in HD109 vs Control in shCTRL lines.

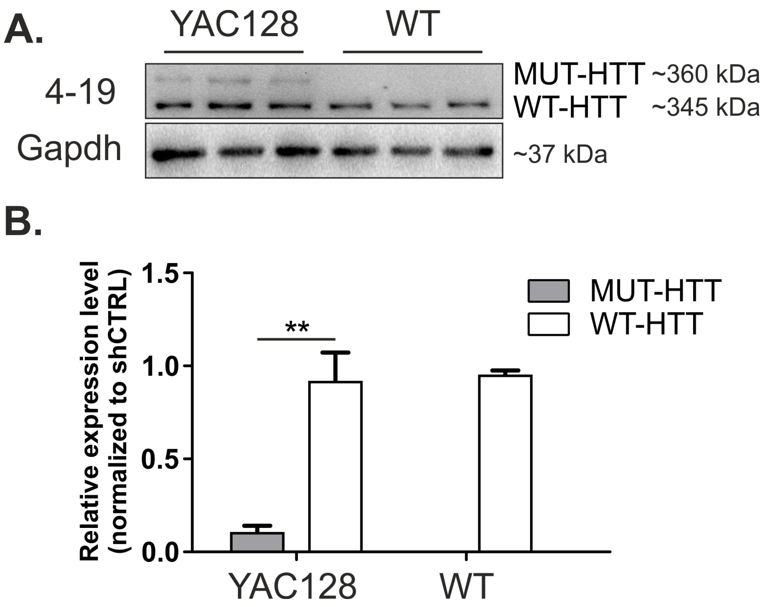

Figure S5: Human mutant (MUT) and mouse wild-type (WT) HTT protein expression in YAC128 and WT iPSC analyzed with western blot using antibody specific to N-terminal part of HTT (aminoacids 4-19). The presence of mutant HTT does not influence level of wild-type HTT and mutant HTT expression is approximately 9 times lower than mouse HTT in YAC128 iPSC.

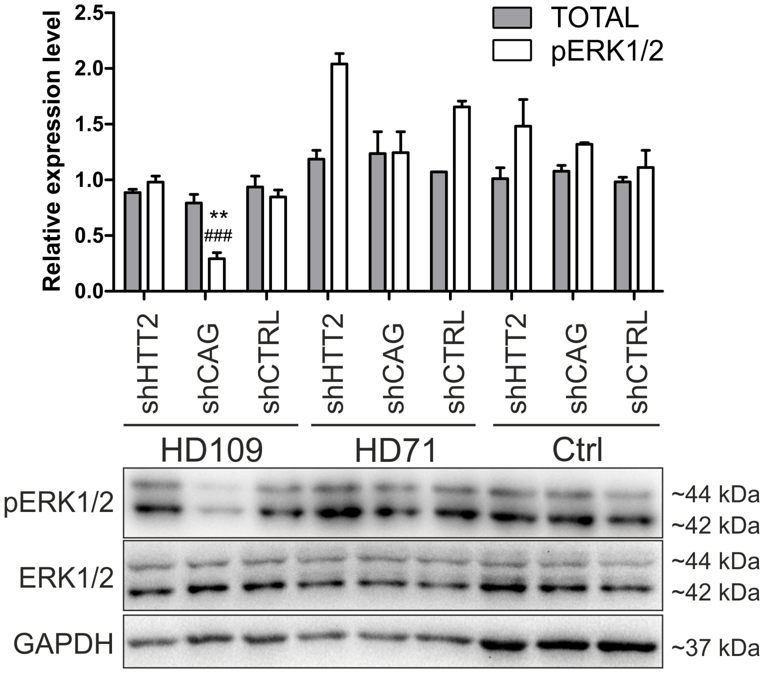

Figure S6: Effects of mutant HTT knockdown on Wnt pathway in human iPSCs. We observed no effects on total or phospho-ERK1/2 (Thr202/Tyr204) human shRNA iPSC lines. * - statistically significant difference vs isogenic shCTRL line; # - statistically significant difference vs Ctrl shCTRL line; ** and ## $p<0.01$; *** and ### $p<0.001$. For each patient, one cell line per each reagent was used

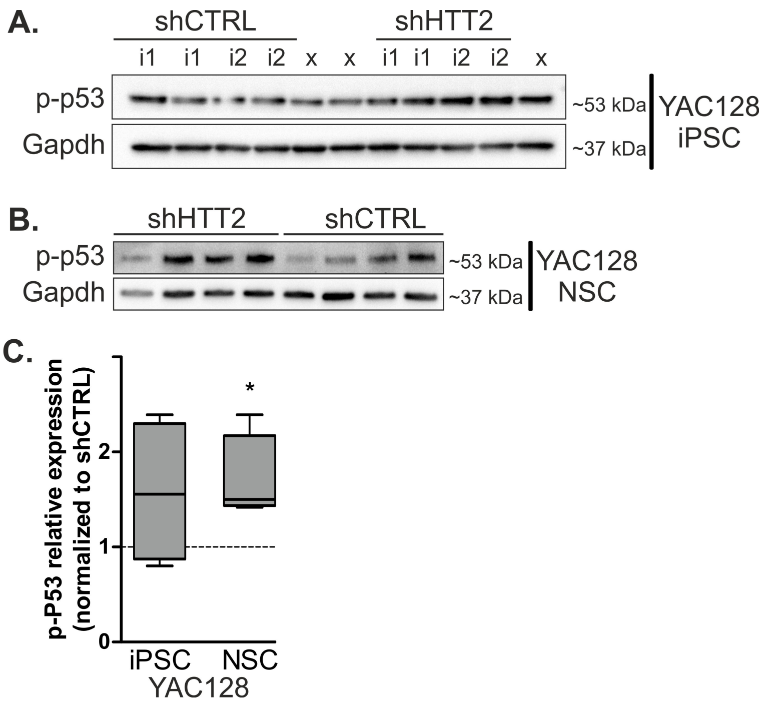

Figure S7: p53 phosphorylated at serine 15 is affected in shHTT2 iPSC (A, C) and NSC (B, C) lines, similarly to total p53. *$p<0.05$

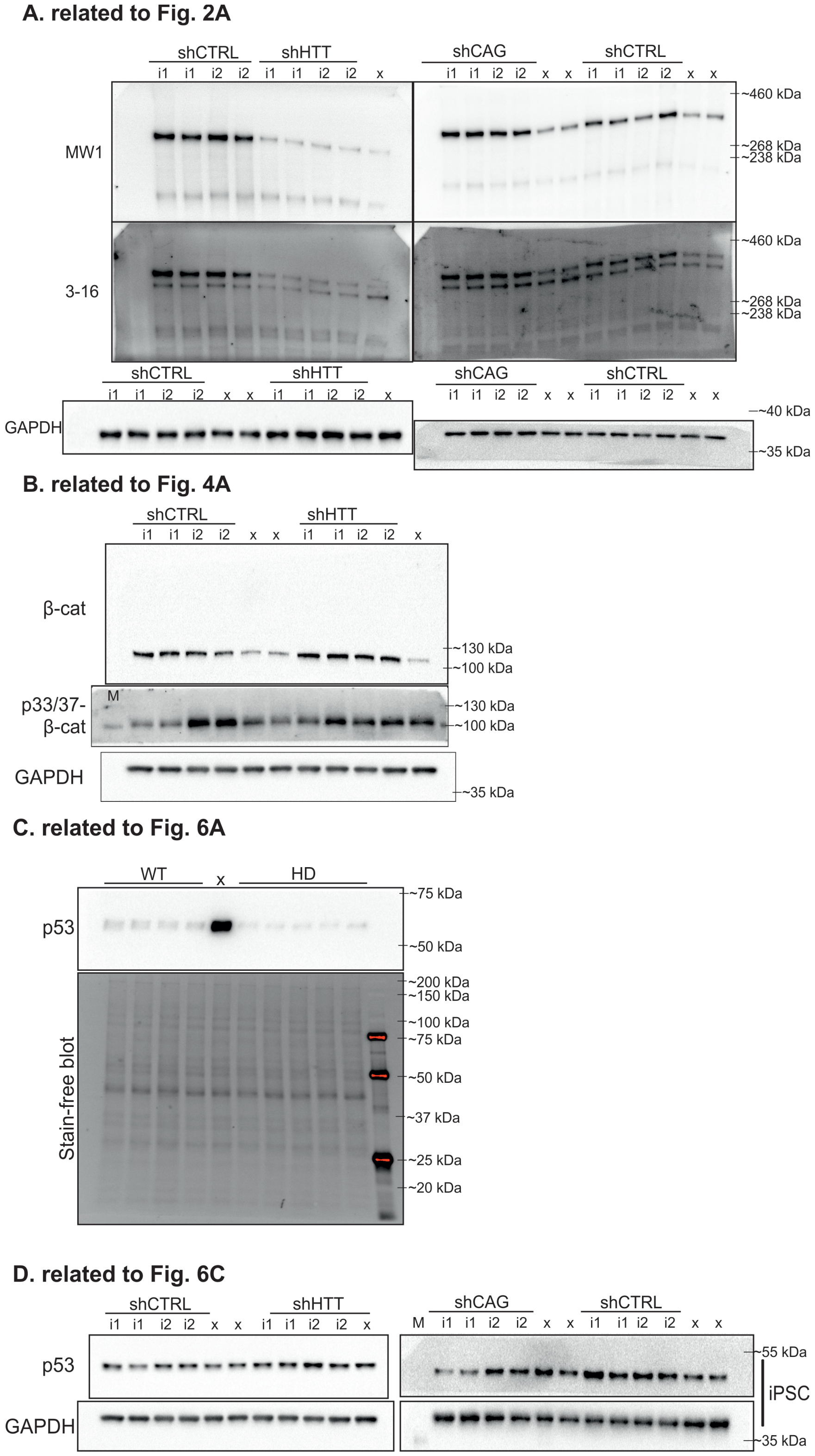

Figure S8: Full-length blots for blots with cropped lanes in the main text figures, i.e. Fig. 2B (A), Fig. 4A (B), Fig. 6A (C) and C (D). Note, that whole-gel blots were cut into pieces to simultaneously stain for various proteins of different molecular weights. Lanes marked with X- were removed from main text figures; these lanes are positive controls and calibrator samples. M – protein ladder lane.
